# The Nutrient-Responsive Hormone CCHamide-2 Controls Growth by Regulating Insulin-like Peptides in the Brain of *Drosophila melanogaster*

**DOI:** 10.1371/journal.pgen.1005209

**Published:** 2015-05-28

**Authors:** Hiroko Sano, Akira Nakamura, Michael J. Texada, James W. Truman, Hiroshi Ishimoto, Azusa Kamikouchi, Yutaka Nibu, Kazuhiko Kume, Takanori Ida, Masayasu Kojima

**Affiliations:** 1 Department of Molecular Genetics, Institute of Life Science, Kurume University, Kurume, Fukuoka, Japan; 2 Department of Germline Development, Institute of Molecular Embryology and Genetics, Kumamoto University, Kumamoto, Japan; 3 Janelia Research Campus, Howard Hughes Medical Institute, Ashburn, Virginia, United States of America; 4 Graduate School of Science, Nagoya University, Nagoya, Aichi, Japan; 5 Precursory Research for Embryonic Science and Technology, Japan Science and Technology Agency, Tokyo, Japan; 6 Department of Cell and Developmental Biology, Weill Medical College of Cornell University, New York, New York, United States of America; 7 Graduate School of Pharmaceutical Sciences, Nagoya City University, Nagoya, Aichi, Japan; 8 Division for Searching and Identification of Bioactive Peptides, Department of Bioactive Peptides, Frontier Science Research Center, University of Miyazaki, Miyazaki, Miyazaki, Japan; Washington University Medical School, UNITED STATES

## Abstract

The coordination of growth with nutritional status is essential for proper development and physiology. Nutritional information is mostly perceived by peripheral organs before being relayed to the brain, which modulates physiological responses. Hormonal signaling ensures this organ-to-organ communication, and the failure of endocrine regulation in humans can cause diseases including obesity and diabetes. In *Drosophila melanogaster*, the fat body (adipose tissue) has been suggested to play an important role in coupling growth with nutritional status. Here, we show that the peripheral tissue-derived peptide hormone CCHamide-2 (CCHa2) acts as a nutrient-dependent regulator of *Drosophila* insulin-like peptides (Dilps). A BAC-based transgenic reporter revealed strong expression of CCHa2 receptor (CCHa2-R) in insulin-producing cells (IPCs) in the brain. Calcium imaging of brain explants and IPC-specific *CCHa2-R* knockdown demonstrated that peripheral-tissue derived CCHa2 directly activates IPCs. Interestingly, genetic disruption of either *CCHa2* or *CCHa2-R* caused almost identical defects in larval growth and developmental timing. Consistent with these phenotypes, the expression of *dilp5*, and the release of both Dilp2 and Dilp5, were severely reduced. Furthermore, transcription of *CCHa2* is altered in response to nutritional levels, particularly of glucose. These findings demonstrate that CCHa2 and CCHa2-R form a direct link between peripheral tissues and the brain, and that this pathway is essential for the coordination of systemic growth with nutritional availability. A mammalian homologue of CCHa2-R, Bombesin receptor subtype-3 (Brs3), is an orphan receptor that is expressed in the islet β-cells; however, the role of Brs3 in insulin regulation remains elusive. Our genetic approach in *Drosophila melanogaster* provides the first evidence, to our knowledge, that bombesin receptor signaling with its endogenous ligand promotes insulin production.

## Author Summary

Animals need to couple growth with nutritional availability for proper development and physiology, which leads to better survival. Nutritional information is mostly perceived by peripheral organs, particularly metabolic organs such as adipose tissue and gut, before being relayed to the brain, which modulates physiological responses. Hormonal signaling ensures this organ-to-organ communication, and defects in this endocrine regulation in humans often cause diseases including obesity and diabetes. In the fruit fly *Drosophila melanogaster*, adipose tissue (the “fat body”) has been suggested to play an important role in coordinating growth with metabolism. Here, we show that the *Drosophila CCHamide-2 (CCHa2)* gene, expressed in the fat body and gut, encodes a nutrient-sensitive peptide hormone. The CCHa2 peptide signals to neuroendocrine cells in the brain that produce *Drosophila* insulin-like peptides (Dilps) through its receptor (CCHa2-R) and promotes the production of Dilps. Mutants of both *CCHa2* and *CCHa2-R* display severe growth retardation during larval stages. These results suggest that CCHa2 and CCHa2-R functionally connect peripheral tissues with the brain, and that CCHa2/CCHa2-R signaling coordinates the animal’s growth with its nutritional conditions by regulating its production of insulin-like peptides.

## Introduction

Organisms need to coordinate growth and metabolism with their nutritional status to ensure proper development and the maintenance of homeostasis. In multicellular animals, nutritional information is mostly perceived by peripheral organs. It is subsequently relayed to other peripheral organs or to the central nervous system (CNS), which generates appropriate physiological and behavioral responses. Endocrine systems ensure this type of organ-to-organ communication *via* hormonal signals secreted from specialized glandular cells. For example, mammalian insulin is secreted from pancreatic β-cells in response to high blood glucose levels; insulin is then received by its receptor in the liver as well as in many other tissues to promote glucose uptake and anabolism, thereby reducing blood sugar levels [1]. In a similar manner, leptin secreted from adipose tissues is received by the hypothalamus, where it acts to alter energy expenditure and food intake [2] [3] [4] [5]. Caloric restriction reduces the secretion of leptin, leading to both an increase in appetite and a decrease in energy expenditure, which is known to be an adaptive response to starvation [6]. These findings demonstrate the significance of peripheral tissues in the maintenance of homoeostasis [7]. However, only a few peripheral hormones have been identified, and the mechanisms by which they regulate an organism's development or physiology in response to external stimuli remain elusive.

It has been reported that the endocrine system of *Drosophila melanogaster* allows adipose tissue, known as the fat body, to communicate with the CNS in a manner similar to that observed in mammals. This signaling depends on nutritional conditions and ultimately couples growth and metabolism with nutritional status. To date, two pathways have been described. In one pathway described from larvae, the fat body-specific down-regulation of either the Slimfast (Slif) amino acid transporter or the Target of Rapamycin (TOR) nutrient-sensing pathway affects systemic growth, suggesting that a hitherto unidentified amino acid-dependent signal(s) is secreted by the fat body for proper growth control [8]. In a second pathway that was identified in adults, Unpaired-2 (Upd2), which is a functional analogue of leptin, was identified as another fat body-derived growth regulator [9]. The expression of *upd2* is both sugar- and lipid-sensitive and is apparently independent of the amino acid-activated TOR pathway [9]. Although no signaling molecules that act downstream of the Slif/TOR pathway have been identified yet, these fat body-derived signals ultimately regulate the production of insulin-like peptides (*Drosophila* insulin-like peptides; Dilps) secreted from the brain [10] [9].

Dilps are evolutionarily conserved peptide hormones with functions similar to those of mammalian insulin/insulin-like growth factor (IGF), including the control of tissue growth and blood sugar levels in response to nutritional conditions [11,12] [13] [14] [15] [16]. Eight *dilp* genes exist in the *Drosophila melanogaster* genome [11] [15] [16]. Unlike mammalian insulin, which is secreted from the pancreas, the major Dilps (Dilp2, -3, and -5) are specifically expressed in bilateral clusters of neurosecretory cells [insulin-producing cells (IPCs)] located in the anteromedial region of the brain hemispheres [11] [12]. With regard to the regulation of insulin-like peptides, the knockdown of the Slif/TOR pathway or *upd2* in the larval fat body results in the down-regulation of Dilp2 secretion [9,10]. Upd2, a type-I cytokine, activates the JAK/STAT pathway through its receptor Domeless (Dome) [17] [18]. Dome is expressed in the GABAergic neurons juxtaposed to the IPCs in the adult brain. Activation of Dome by Upd2 blocks GABAergic inhibition of the IPCs and thereby facilitates Dilp secretion [9]. Therefore, signaling from peripheral tissues to the brain appears to be essential for the regulation of organismal growth and metabolism in response to nutrition availability in *Drosophila melanogaster*.

In this study, we investigated the roles of CCHa2 and its receptor in growth control in *Drosophila melanogaster*. CCHa2 was identified as a bioactive peptide that activates a G protein-coupled receptor (GPCR) encoded by *CG14593* (now named *CCHa2-R*) [19]. Strong expression of CCHa2 in the larval fat body and gut motivated us to examine the roles of CCHa2 and its receptor in nutrient sensing and growth control. By generating mutants of *CCHa2* and *CCHa2-R*, we show that CCHa2/CCHa2-R signaling from the periphery to the CNS can control the synthesis and secretion of Dilps. Our results demonstrate that CCHa2 is a novel hormone derived from peripheral tissues and that CCHa2/CCHa2-R form an additional afferent hormonal signaling pathway that coordinates systemic growth with nutrition availability.

## Results

### CCHa2 is a selective nutrition-sensitive hormone derived from peripheral tissues

**Fig 1:**
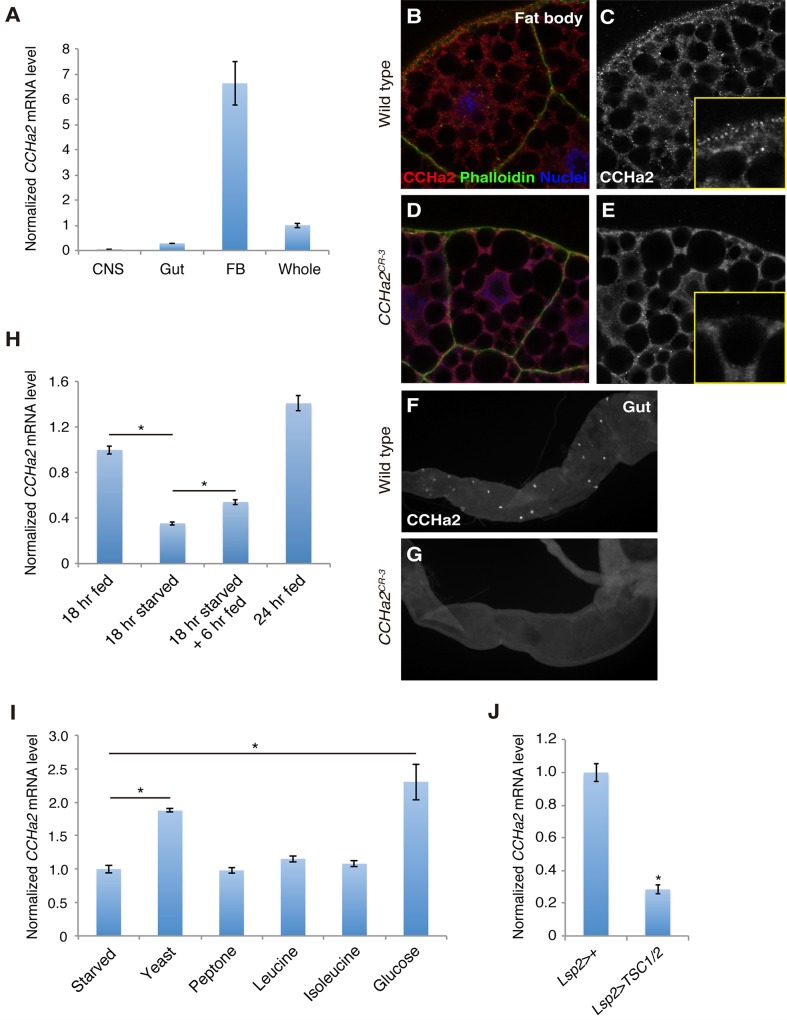
*CCHa2* is a selective nutrient-sensitive hormone derived from peripheral tissues. (A) RT-qPCR was performed on RNA extracted from larval tissues. *CCHa2* is predominantly expressed in the larval fat body. (B-G) Anti-CCHa2 immunostaining of the fat body (B-E) and gut (F, G) from wild-type and *CCHa2* mutants. Insets in C and E are high-magnification images of the rim of the fat cell. The punctate staining in B and C that is absent from D and E is the CCHa2 signal; the dimmer uniform signal is non-specific background staining, since it also appears in the tissue from *CCHa2*-null animals (E). (H) Effects of nutritional status on *CCHa2* expression. Third-instar larvae were raised under the indicated nutritional conditions, and *CCHa2* mRNA levels were tested by RT-qPCR using whole-animal RNA extracts. *CCHa2* expression was decreased by starvation and recovered by re-feeding the starved larvae with yeast paste. (I) Nutrient requirements for *CCHa2* expression. Third-instar larvae starved for 18 hours were re-fed with the indicated nutrients for 6 hours, and *CCHa2* mRNA levels in whole animals were measured by RT-qPCR. Yeast and glucose significantly promoted *CCHa2* expression. (J) Effects of fat-body-specific TOR pathway manipulation on *CCHa2* expression. The TOR pathway was inhibited by expressing *TSC1/2* in the fat body using *Lsp2-GAL4*. *CCHa2* mRNA levels in whole animals were measured by RT-qPCR.

We first measured the expression of *CCHa2* mRNA in larval tissues using quantitative RT-PCR (RT-qPCR). As shown in Fig 1A, *CCHa2* was predominantly detected in the fat body and the gut, with only very low expression detected in the CNS. We also examined CCHa2 expression using CCHa2 antisera, which detected punctate staining in the cytoplasm of the fat cells (Fig 1B and 1C). CCHa2 immunoreactivity was also detected in endocrine cells in the gut as previously reported [20] (Fig 1F). These signals were specific for CCHa2, because they were absent in *CCHa2* mutants (Fig 1D and 1E and 1G).

We next examined the nutritional dependence of *CCHa2* expression. In this assay, third-instar larvae [72 hours after egg laying (AEL)] were starved for 18 hours on water agar plates, and the relative amount of *CCHa2* mRNA in the whole animal was quantified. After starvation, the expression of *CCHa2* was significantly decreased, but levels recovered when the larvae were re-fed with yeast paste (Fig 1H).

To determine which nutrient signal controls *CCHa2* expression, larvae were re-fed with different substances after starvation. Interestingly, both yeast and glucose induced *CCHa2* expression (Fig 1I). It has been reported that the TOR nutrition-sensing pathway is activated by amino acids but not by glucose [10]. Nonetheless, we tested the involvement of this pathway in *CCHa2* regulation in the fat body. When the TOR pathway was blocked in the fat body by the overexpression of signaling components *TSC1/2*, *CCHa2* expression was significantly reduced (Fig 1J). (It should be noted that the knockdown of the TOR pathway in the fat body severely affects larval growth [8]; therefore, *Lsp2-GAL4*, which is expressed in the fat body at the wandering stage – after the growth period [13] – was used to overexpress *TSC1/2* in order to avoid secondary effects from a systemic growth defect.) These observations suggest that *CCHa2* expression is responsive to glucose and the TOR pathway. Given that glucose alone is sufficient to promote *CCHa2* expression, CCHa2 appears to be distinct from the currently unidentified fat body-derived factor previously proposed to act downstream of the Slif/TOR pathway [10].

### The receptor for *CCHa2* is expressed in the brain

**Fig 2:**
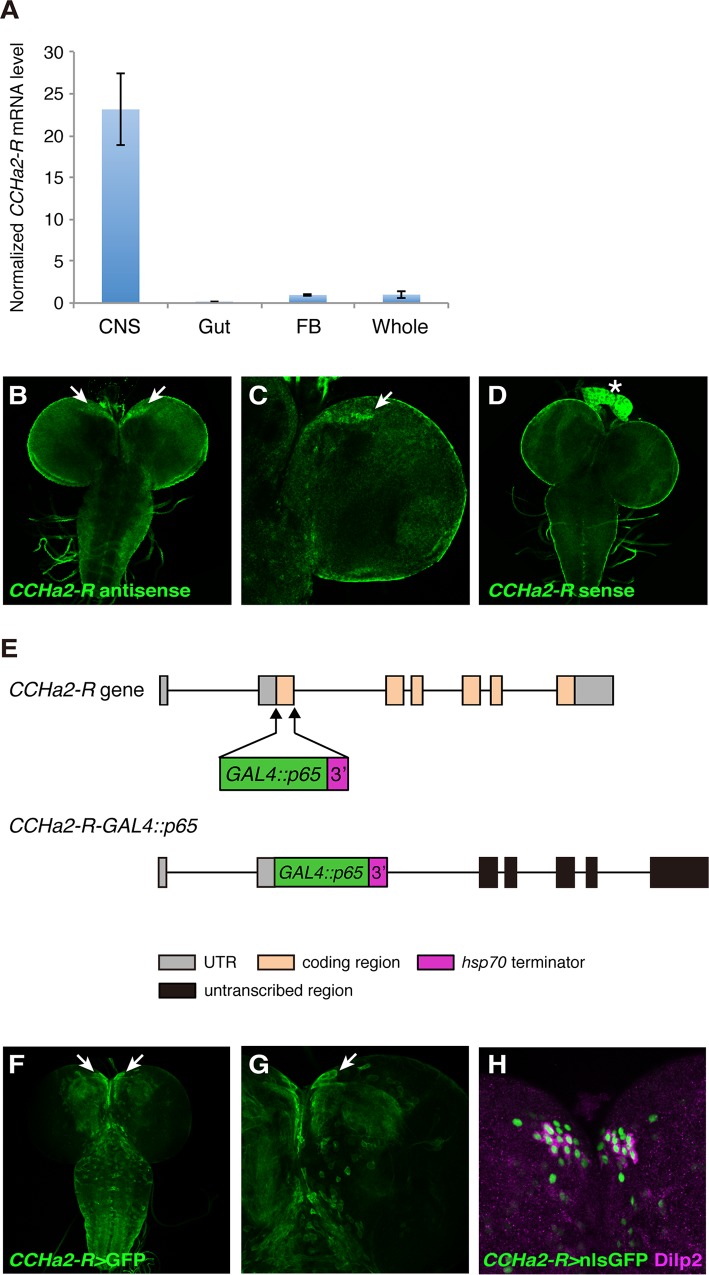
The CCHa2 receptor is expressed in the brain including the IPCs. (A) RT-qPCR was performed on RNA extracted from larval tissues. *CCHa2-R* is specifically expressed in the CNS. (B-D) *CCHa2-R* mRNA was detected by *in situ* hybridization; signals were amplified by the TSA system. *CCHa2-R* mRNA is expressed in cells located in the anteromedial region of the brain (arrows in B, C). (D) Sense probe control for B and C. Signals in the ring gland (asterisk) and the CNS surface are non-specific background staining. (E) Schematic drawing of the *CCHa2-R-GAL4*::*p65* construct. (F-G) Expression of membrane-targeted GFP driven by *CCHa2-R-GAL4*::*p65*. GFP was detected in the anteromedial cells (arrows). (H) Nuclear-localized GFP driven by *CCHa2-R-GAL4*::*p65* is present in all the IPCs, which are marked with anti-Dilp2. Neighboring GFP-labeled cells express NPF or SIFamide (S1 Fig).

We performed RT-qPCR assays to examine the larval expression pattern of *CCHa2-R (CG14593)*, which encodes the receptor for CCHa2. As shown in Fig 2A, *CCHa2-R* mRNA was detected specifically in the larval CNS. Fluorescent *in situ* hybridization (FISH) of larval brains with a *CCHa2-R* antisense RNA probe yielded signal in a subset of cells located at the anteromedial region of the brain (Fig 2B and 2C), which contains several types of neuroendocrine cells, including the IPCs [21]. To increase sensitivity for the mapping of *CCHa2-R* expression, its pattern was indirectly visualized using a GAL4 construct that recapitulates endogenous *CCHa2-R* expression. For this purpose, we established a transgenic fly line that carries an ~80-kb genomic region containing the entire *CCHa2-R* coding region, as well as substantial flanking sequence. The coding portion of the first CCHa2-R coding exon was replaced with sequence encoding the strong GAL4::p65 transcriptional activator (Fig 2E; *CCHa2-R*-*GAL4*::*p65*). Consistent with the endogenous expression pattern (Fig 2B and 2C), strong GFP signals were detected in the anteromedial region of the brain in transgenic flies in which *CCHa2-R*-*GAL4*::*p65* drove the expression of *UAS-nls*::*GFP* (*CCHa2-R>nlsGFP*) (Fig 2F and 2G). Low levels of GFP expression were also observed in a number of cells in the brain and the ventral nerve cord (VNC) (Fig 2F and 2G), in which no *CCHa2-R* expression was detected by FISH, probably due to its lower sensitivity. When the transgenic larval brains were co-stained with anti-Dilp2 antibody, CCHa2-R>nlsGFP colocalized with the Dilp2 immunostaining (Fig 2H). We also found that neighboring peptidergic neurons that contain neuropeptide F (NPF) and SIFamide (SIFa), both also showed CCHa2-R>nlsGFP-expression (S1 Fig). Thus, *CCHa2-R* is expressed in a few types of neuroendocrine cells including the IPCs.

### *CCHa2-R* mutations affect the production of insulin-like peptides in the brain

**Fig 3:**
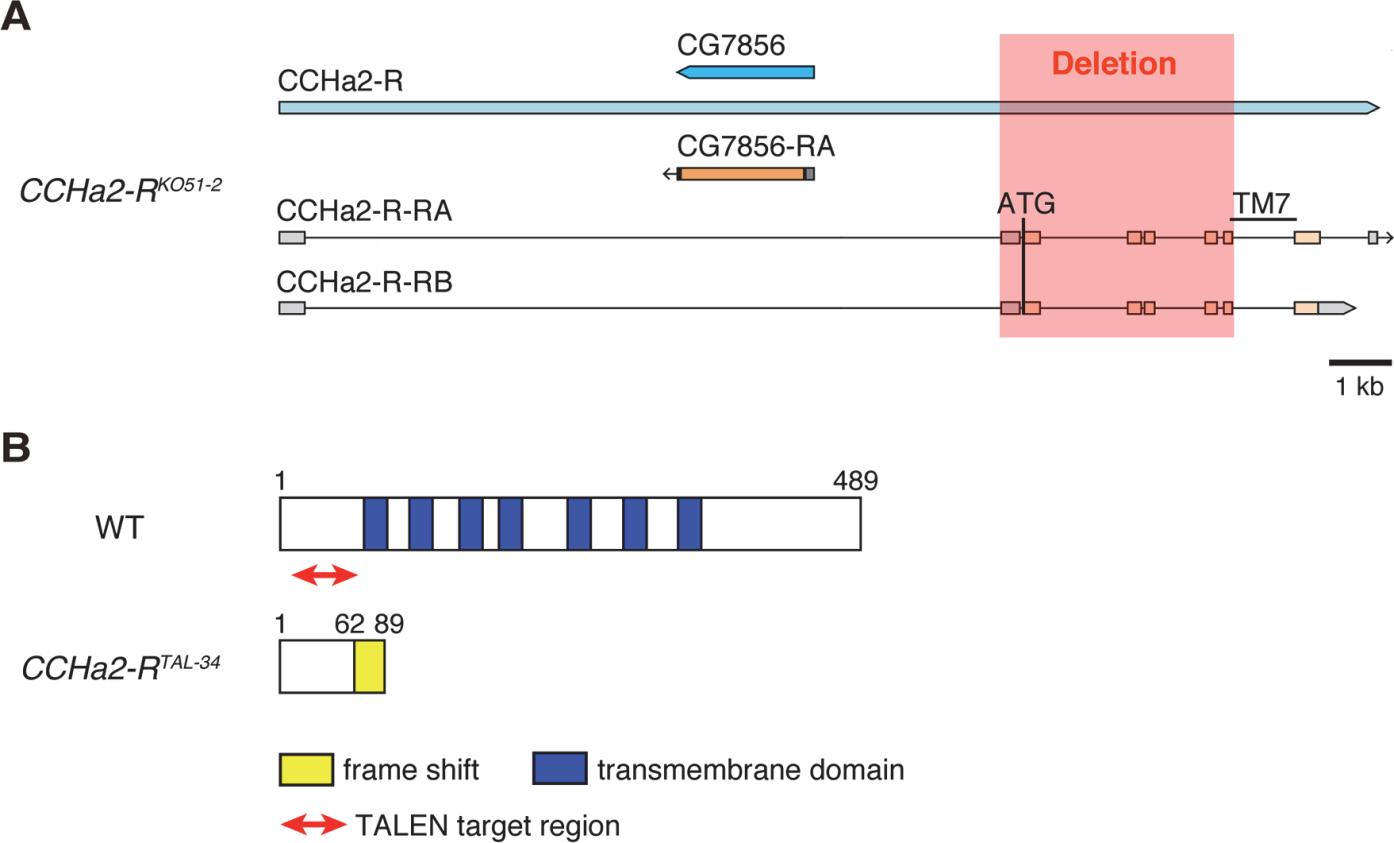
Generation of *CCHa2-R* mutants. (A) The *CCHa2-R* ^*KO51-2*^ allele was generated by gene targeting using homologous recombination. In *CCHa2-R* ^*KO51-2*^, a 4-kb deletion spanning exons 2 through 6 removes a region from the initiating methionine through the middle of 7th transmembrane domain. (B) The *CCHa2-R* ^*TAL-34*^ allele was created using TALENs. A 74-bp deletion was created in the first coding exon of the *CCHa2-R* gene, resulting in a frame-shift at amino acid position 62 and a truncation of the protein.

Since *CCHa2-R* is expressed in the IPCs, we examined whether CCHa2/CCHa2-R signaling is involved in insulin regulation. We generated two mutant *CCHa2-R* alleles, both expected to be nulls. Using gene targeting by homologous recombination [22] [23], most of the *CCHa2-R* coding region – from the translation-initiating methionine through the middle of the 7th transmembrane domain of the encoded GPCR – was deleted, generating the *CCHa2-R* ^*KO51-2*^ allele (Figs 3A and S2A). In a second scheme, transcription activator-like effector nucleases (TALENs) were targeted to sequences near the translation-initiation site of CCHa2-R to create a frame-shift mutation in the *CCHa2-R* coding region [24]. By injecting pairs of TALEN-encoding mRNAs into wild-type embryos, we generated the *CCHa2-R* ^*TAL-34*^ frameshift allele (Figs 3B and S2B and S2C). Although these two mutant alleles are both homozygous-viable, we used them in transheterozygous combination (*i*.*e*., *CCHa2-R* ^*KO51-2*^/*CCHa2-R* ^*TAL-34*^) to avoid the effects of any unexpected secondary mutations.

**Fig 4:**
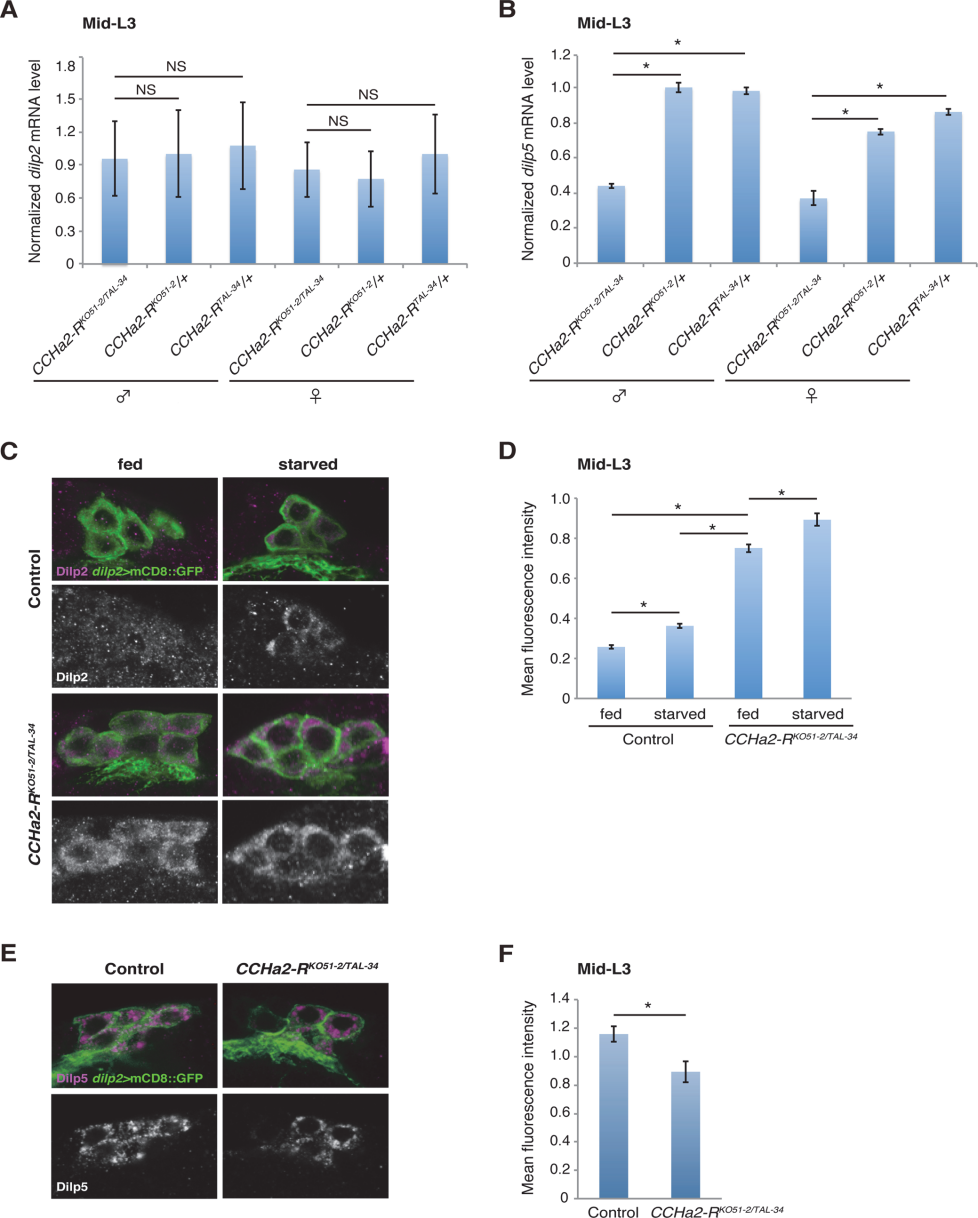
*CCHa2-R* mutations affect the production of Dilp2 and Dilp5 in IPCs. Transcript and protein levels of brain Dilps were examined in mid-third-instar larvae (96 hours AEL). (A) Relative expression level of *dilp2* mRNA in whole larval RNA extracts was quantified by RT-qPCR. Transcription of *dilp2* was unaffected by the loss of *CCHa2-R*. (B) Relative expression level of *dilp5* mRNA in whole larval RNA extracts was quantified by RT-qPCR. Expression of *dilp5* was significantly reduced in *CCHa2-R* mutants. (C) Dilp2 in the IPCs was detected by Dilp2 antibody in wild-type and *CCHa2-R* mutants in which the IPCs were labeled by *dilp2-GAL4*-driven *UAS-mCD8*::*GFP*. Wild-type and *CCHa2-R* mutant larvae were either fed or starved for 24 hours before staining. (D) Dilp2 levels in the IPCs were quantified. Fluorescence intensities for Dilp2 were normalized against those for GFP on the same confocal section (n = 52, 48, 51, 45 cells for fed control, starved control, fed mutants, and starved mutants, respectively). (E) Dilp5 in the IPCs was detected by anti-Dilp5 antibody in wild-type and *CCHa2-R* mutants, in which the IPCs were labeled by *dilp2-GAL4* driving *UAS-mCD8*::*GFP*. (F) Dilp5 levels in the IPCs were quantified (n = 37 and 36 for fed control and fed mutants, respectively).

These mutant alleles were first used to examine whether *CCHa2-R* is required for the transcription of two *Drosophila* insulin-like peptide (*dilp*) genes. It has been reported that *dilp2*, *dilp3*, and *dilp5* are expressed in IPCs [11] [12]. As previously reported, the expression level of *dilp3* is very low during the growth period (S3A Fig) [13] [14], and neither growth nor developmental timing is altered in *dilp3*-null mutants [25]. Nonetheless, we tested whether *dilp3* is under the control of CCHa2-R signaling. As shown in S3B Fig, *dilp3* mRNA levels were not altered by *CCHa2-R* mutations. In contrast, *dilp2* and *dilp5* are highly expressed in feeding larvae and have substantial influence over larval growth [13] [14] [25]. Therefore, we focused on these two Dilps. For this analysis, total RNA extracted from whole larvae was used, as *dilp2* and *dilp5* are predominantly expressed in the CNS during the larval stages examined [26,27]. In mid-L3 (96 hours AEL) larvae, *dilp2* expression was not significantly altered by the loss of *CCHa2-R* (Fig 4A). In contrast, the expression of *dilp5* mRNA was remarkably reduced in *CCHa2-R* mutant larvae, compared to control larvae heterozygous for either the *CCHa2-R* ^*KO51-2*^ or *CCHa2-R* ^*TAL-34*^ allele, regardless of gender (Fig 4B). It was reported that starvation or Slif/TOR inhibition down-regulates the secretion of Dilp2, but not its transcription [28] [10]. We therefore examined the protein levels of Dilp2 in IPCs by staining larval brains with anti-Dilp2. It has been shown previously that, when *dilp2* transcription is constant, increased Dilp2 within the cytoplasm of IPCs reflects decreased Dilp2 release into the hemolymph, and intracellular Dilp2 has been used as a sign of Dilp2 retention in the IPCs [10]. Consistent with this, stronger Dilp2 signals were observed in the IPCs of starved wild-type larvae than in those under fed conditions (Fig 4C). When *CCHa2-R* mutant larvae were fed, their IPCs showed increased Dilp2 immunoreactivity, which was much stronger than that observed in starved or fed control larvae (Fig 4C). To accurately compare the signals, the Dilp2 signal intensity in the IPCs for each condition was normalized against signals of membrane-bound GFP expressed under the control of the *dilp2-GAL4* driver as an internal control (because, as noted above, *dilp2* transcription is unaltered in the mutants). The quantification results clearly show that levels of Dilp2 protein in the mutant IPCs were significantly higher than those in the wild type (Fig 4D). Dilp5 protein levels were also quantified by the same methods in wild-type and *CCHa2-R* mutant IPCs. Despite a ~40% reduction in *dilp5* mRNA in the mutant IPCs (Fig 4B), Dilp5 protein levels only dropped by about 20%, suggesting decreased release (Fig 4E and 4F). These results suggest that the secretion of both Dilp2 and Dilp5 is severely affected by the loss of *CCHa2-R*. We also noticed that Dilp2 levels in mutant IPCs under starved conditions were slightly higher than those under fed conditions (Fig 4C and 4D), suggesting that *CCHa2-R* is not the sole nutrient-sensitive Dilp regulator. Analysis of feeding activity using dyed yeast demonstrated no significant differences in dye ingestion between wild-type and *CCHa2-R* mutants (S4 Fig), suggesting that down-regulation of CCHa2/CCHa2-R signaling does not affect larval feeding behaviors. Taken together, these results show that *CCHa2-R* plays a crucial role in regulating the synthesis and secretion of insulin-like peptides in IPCs.

### Peripheral tissue-derived CCHa2 induces *dilp5* expression in the brain

**Fig 5:**
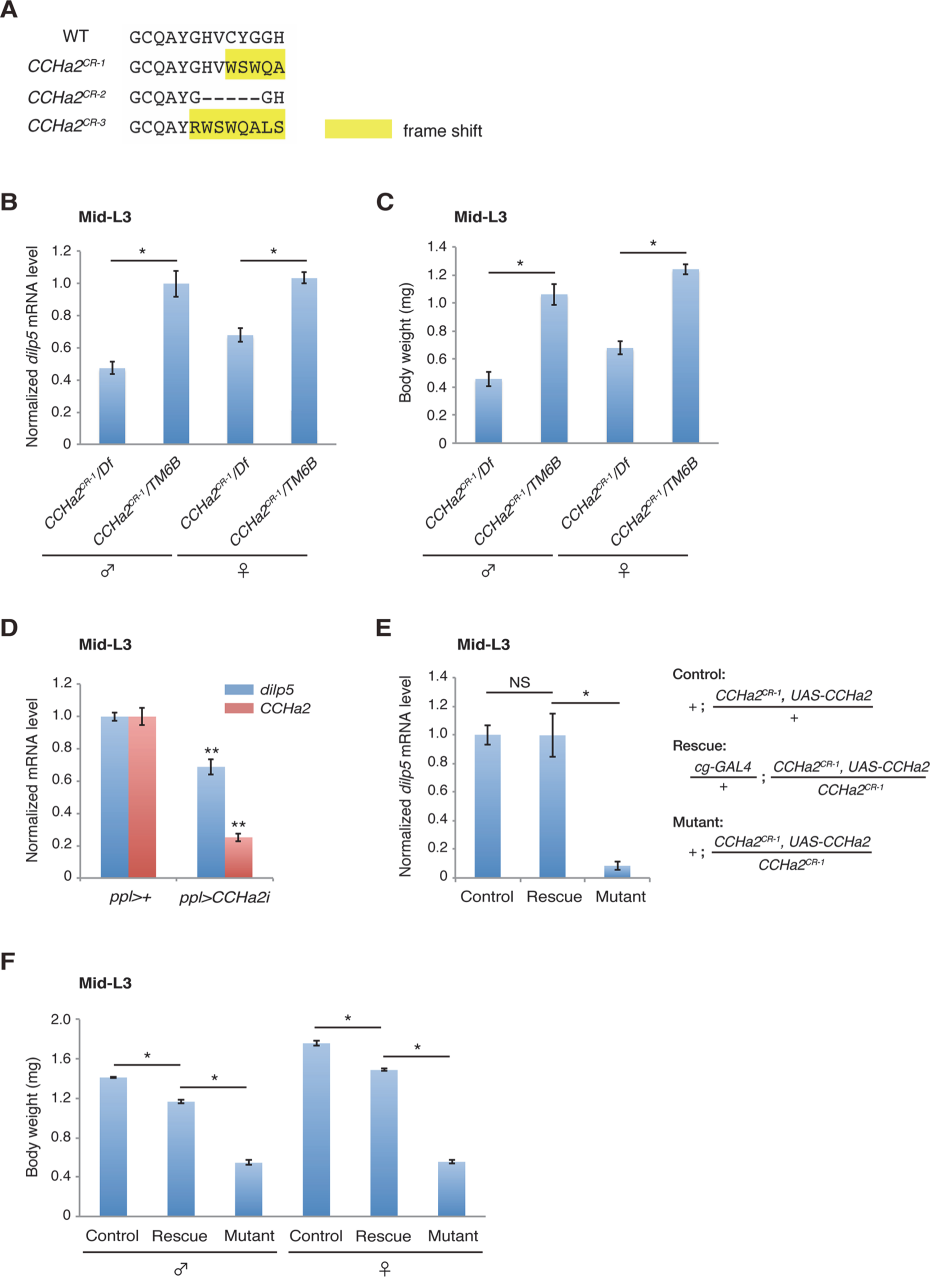
Peripheral tissue-derived CCHa2 induces *dilp5* expression in the brain. (A) Amino acid sequences of the CCHa2 peptide formed in the *CCHa2* mutant alleles. (B) *dilp5* mRNA levels in mid-third-instar larvae (96 hours AEL) were quantified by RT-qPCR using whole larval RNA extracts. *dilp5* transcription was down-regulated in *CCHa2* mutants. (C) Larval body weight was measured at the mid-third-instar larval stage (96 hours AEL; 5 to 10 larvae per batch, n = 3 batches). *CCHa2* mutant larvae weighed significantly less than wild-types. (D) *CCHa2* was knocked down in the fat body and gut using the *ppl-GAL4* driver, and *dilp5* mRNA levels were quantified by RT-qPCR. *dilp5* expression was significantly reduced in the *CCHa2*-knockdown larvae. (E) *CCHa2* was over-expressed in fat bodies of *CCHa2* mutant animals using the *cg-GAL4* driver, and *dilp5* mRNA levels in the CNS were quantified by RT-qPCR. *dilp5* transcription was completely restored by *CCHa2* expression in the fat body. (F) The body weight of rescued animals (96 hours AEL; 5 to 10 larvae per batch, n = 3 batches). Body weight was mostly rescued by *CCHa2* expression in the fat body.

To examine whether CCHa2 acts together with CCHa2-R in the control of Dilps *in vivo*, we generated animals carrying a defective *CCHa2* gene using the CRISPR/Cas system [29]. Short guide RNA was targeted to the sequence corresponding to the CCHa2 peptide-coding region, resulting in the isolation of putative null alleles for *CCHa2* (*CCHa2* ^*CR-1*^, *CCHa2* ^*CR-2*^, and *CCHa2* ^*CR-3*^) (Figs 5A and S5). All of these alleles displayed virtually the same phenotypes, so the results for *CCHa2* ^*CR-1*^ are shown as representative. First, RT-qPCR analyses showed that the expression of *dilp5* mRNA was significantly reduced in *CCHa2* ^*CR-1*^ */Df* hemizygotes compared to heterozygous control larvae regardless of sex (Fig 5B). These results are similar to those seen in *CCHa2-R* mutants (compare Figs 4B and 5B). Consistent with these reduced *dilp5* levels, the body weight of mid-third-instar larvae (96 hours AEL) was markedly lower in the *CCHa2* hemizygotes than in the heterozygous control regardless of sex (Fig 5C). These results suggest that CCHa2 and CCHa2-R act together to regulate *dilp5* expression in the brain.

To clarify whether peripheral tissues are responsible for the CCHa2-dependent regulation of *dilps* in the brain, *CCHa2* was specifically knocked down in the fat body and gut using targeted RNAi driven by the *ppl-GAL4* driver [8] [30]. As shown in Fig 5D, *dilp5* mRNA levels were significantly reduced in *CCHa2*-knockdown larvae, indicating that peripheral tissue-derived CCHa2 activates *dilp5* expression in the brain. To further confirm the importance of CCHa2 signaling from the periphery to the CNS, we expressed *CCHa2* in the fat body in the *CCHa2* mutant background using the *cg-GAL4* driver [30]. To ensure the detection of brain-specific changes in *dilp5* expression, RNA extracted from the brain was used for RT-qPCR. As shown in Fig 5E, *CCHa2* expression in the fat body completely restored *dilp5* expression in the brain of *CCHa2* mutants. Consistent with the rescued *dilp5* expression, the body weight of these larvae was mostly recovered (Fig 5F). These results demonstrate that CCHa2 released from peripheral tissues controls *dilp5* expression in the brain.

### CCHa2/CCHa2-R signaling forms a direct link between peripheral tissues and the brain

**Fig 6:**
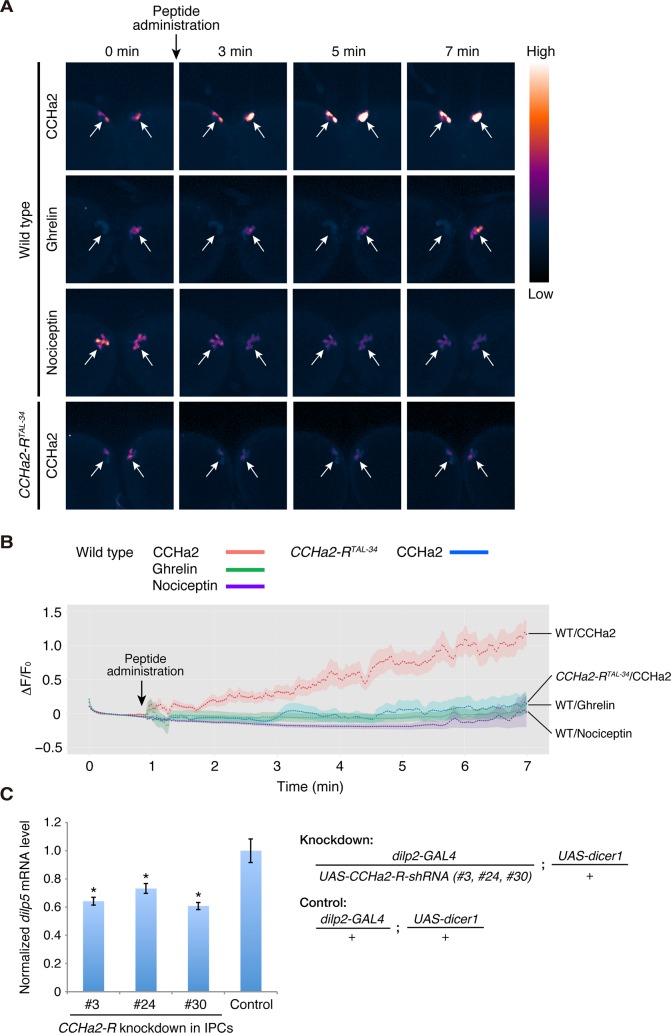
CCHa2 directly activates IPCs in the brain. (A, B) Calcium imaging of larval brain explants. Brains dissected from late-third-instar larvae were exposed to peptide hormones: wild-type (*dilp2-GAL4/UAS-GCaMP6s*) and *CCHa2-R* mutant (*dilp2-GAL4*, *CCHa2-R* ^*TAL-34*^/*UAS-GCaMP6s*, *CCHa2-R* ^*TAL-34*^) brains were challenged with CCHa2; wild-type brains were additionally tested with mammalian ghrelin and nociceptin. GCaMP6s signals in IPCs were measured by confocal microscopy at 4 Hz. Pseudocolored images of selected time points are displayed in (A). ΔF/F_0_ at each time point is plotted in (B). (C) *CCHa2-R* was knocked down specifically in the IPCs using *UAS-CCHa2-R-shRNA* driven by *dilp2-GAL4*, and *dilp5* mRNA levels were quantified by RT-qPCR. Expression of *dilp5* was significantly reduced in the *CCHa2-R*-knockdown larvae.

To clarify whether CCHa2 directly signals to the brain, calcium imaging was performed using *ex vivo* culture of larval brains expressing the fluorescent calcium sensor GCaMP6s [31] in the IPCs. Dissected wild-type or *CCHa2-R* mutant brains were immersed in phosphate buffered saline (PBS). Peptides were added to the culture medium, and fluorescence changes from GCaMP6s were measured by live imaging. As shown in Fig 6A and 6B, signal intensities in wild-type IPCs were dramatically increased upon CCHa2 administration (S1 Movie), indicating that no other tissues are required for relaying the CCHa2 signal to the CNS. In contrast, no such increase in signal was observed in *CCHa2-R* mutant brains upon CCHa2 administration (S2 Movie). The difference in fluorescence intensities between wild-type and *CCHa2-R* mutant IPCs became significant as early as 2 minutes after CCHa2 administration and lasted at least for 7 minutes (S6 Fig). No increase in signal intensity was observed in wild-type brains treated with ghrelin or nociceptin (S3 and S4 Movies, respectively), mammalian peptide hormones which have no homologues in *Drosophila*. These results indicate that the observed activation of the IPCs is a specific response to CCHa2 mediated by CCHa2-R.

To examine whether the CCHa2 signal directly activates the IPCs, rather than being relayed by other neurons, *CCHa2-R* was knocked down specifically in the IPCs. We generated three *UAS-CCHa2-R-shRNA* lines (#3, 24, 30; S7 Fig). When any one of these shRNAs was specifically expressed in the IPCs, *dilp5* mRNA levels were significantly decreased (Fig 6C). These results demonstrate that CCHa2-R is specifically required in IPCs for those cells’ response to the CCHa2 ligand, and suggest that CCHa2 secreted from the peripheral tissues directly signals to IPCs without being relayed by other tissues.

### *CCHa2-R* mutants show defects in larval growth and developmental delay

**Fig 7:**
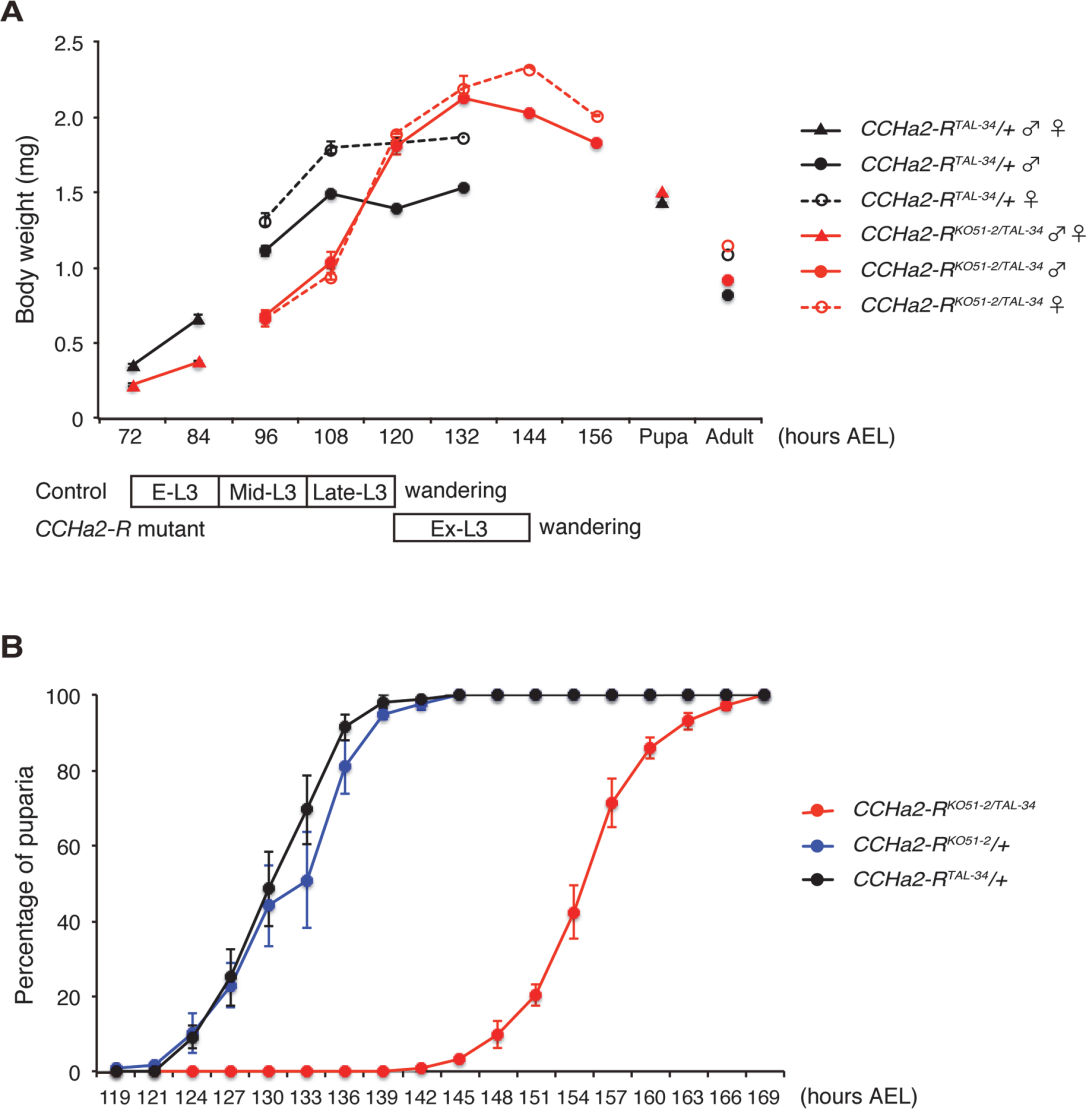
*CCHa2-R* mutants show growth defects and developmental delay. (A) Body weight was measured in batches of 10 to 35 animals, and the average weight per animal was plotted (n = 3 batches). (B) Timing of pupation of 40 animals was examined every 3 hours, and the averages of three experiments were plotted.

Since insulin-like peptides are major growth hormones in flies, we anticipated that abnormal expression and secretion of Dilps in the *CCHa2-R* mutants would lead to growth defects. To test this hypothesis, wild-type and mutant animals were weighed at time points from larval through adult stages. As predicted, *CCHa2-R* transheterozygous mutants weighed markedly less than heterozygous *CCHa2-R* ^*TAL-34/+*^ control larvae from 72 to 108 hours AEL (Fig 7A). However, after 108 hours AEL, the mutant larvae showed rapid weight gain, surpassing wild-type weight at 120 hours AEL (Fig 7A). The body weight of the mutant larvae decreased from 144 hours AEL onward, resulting in pupae and adults of normal weight. In concert with these growth defects, *CCHa2-R* mutants displayed a developmental delay during the larval stages, with the feeding period extended for about 24 hours before entering the wandering stages. This prolonged feeding period resulted in an increase in body weight and delay in pupation and eclosion (Fig 7B). Since broad *dilp2* overexpression caused lethality [11], we were unable to examine whether these developmental delays are a consequence of growth defects or whether developmental timing is regulated independently of growth. These results nevertheless suggest that CCHa2-R is a major growth regulator until around 108 hours AEL, after which time other mechanisms may operate to accelerate the growth of the mutant larvae.

**Fig 8:**
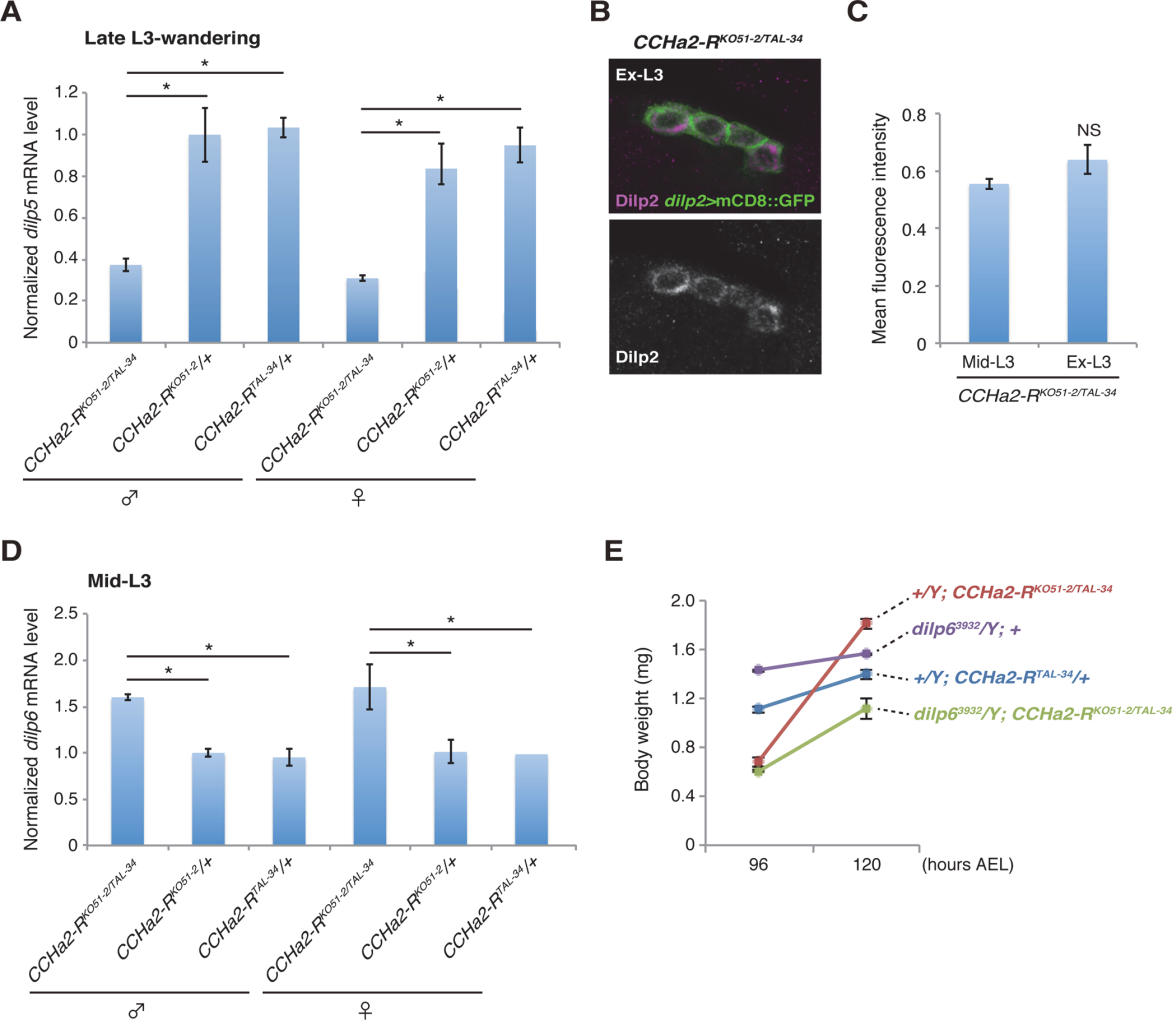
Growth recovery in *CCHa2-R* mutants is due to the up-regulation of *dilp6*. (A) Relative amounts of *dilp5* mRNA were quantified by RT-qPCR using whole animal RNA extracts from larvae at the late third-instar to wandering stages. The *dilp5* mRNA level was significantly reduced in *CCHa2-R* mutants. (B, C) Dilp2 immunoreactivity in the IPCs was compared in *CCHa2-R* mutants at the mid-third instar (Mid-L3) and extended larval stages (Ex-L3). IPCs expressing *dilp2>mCD8*::*GFP* were doubly stained with antibodies against Dilp2 and GFP. Fluorescence intensities of the Dilp2 signals were unchanged in Mid-L3 and Ex-L3 larvae (n = 50 and 28 cells for Mid-L3 and Ex-L3 larvae, respectively). (D) Relative amounts of *dilp6* mRNA were quantified by RT-qPCR using whole-animal RNA extracts from third-instar larvae (Mid-L3, 96 hours AEL). Transcription of *dilp6* was up-regulated in *CCHa2-R* mutants. (E) Body weight of *dilp6*, *CCHa2-R* double mutants and control animals was measured at 96 and 120 hours AEL, and the average weight per animal was plotted (5 to 10 larvae per batch, n = 3 batches).

Both the down-regulation of *dilp5* expression (Fig 8A) and the retention of Dilp2 within the IPCs (Fig 8B and 8C) persisted in the *CCHa2-R* mutants through the last day of the extended larval stage (up to 144 hours AEL). These results support our conclusion that the reduction in Dilp2 and Dilp5 is not the consequence of feeding defects, because these larvae show abnormal Dilp regulation regardless of active feeding and growth. It has been reported that brain-derived Dilps (Dilp2, -3, and -5) are received by an insulin receptor (InR) in the fat body, leading to transcriptional repression of *dilp6* in that tissue [25]. Hence, the reduction in Dilp expression in the brain in *CCHa2-R* mutants could lead to up-regulation of *dilp6* transcription in the fat body. Consistent with this idea, we found that *dilp6* mRNA levels were elevated in *CCHa2-R* mutant larvae (Fig 8D). Furthermore, we found that the removal of *dilp6* from the *CCHa2-R* mutants abolished growth recovery between 96 to 120 hours AEL (Fig 8E). Taken together, these findings indicate that CCHa2/CCHa2-R signaling is a major regulator of Dilps until the mid- to late third-instar larval stages and that the growth recovery observed in later-stage mutants is due to the up-regulation of *dilp6* resulting from the impairment of Dilp expression in the brain.

## Discussion

Some peripheral tissues act as monitors of the nutritional environment and metabolic status. Thus, communication between peripheral organs, particularly from metabolic organs such as adipose tissues or the gut, and the brain is imperative for proper development and the maintenance of homeostasis. Here, we have demonstrated that signaling from peripheral tissues to the CNS mediated by the CCHa2 hormone and its receptor is required for the proper regulation of growth in response to nutritional conditions.

### The CCHa2 peptide is a nutrient-specific Dilp regulator

A previous study suggested the existence of an amino acid-sensitive Dilp regulator(s) in larvae [10]. This as-yet-unidentified Dilp regulator(s) is regulated by the Slif/TOR pathway, and leucine and isoleucine, positive regulators of TOR signaling, are sufficient to promote the secretion of Dilp2 in both *in vivo* and *ex vivo* co-cultures of brain and fat bodies [10]. Our results demonstrated that the TOR pathway is required for *CCHa2* expression during the larval stages (Fig 1J). However, feeding with amino acids, including leucine and isoleucine, was insufficient to promote *CCHa2* expression (Fig 1I). *CCHa2* expression was, however, induced by feeding with glucose (Fig 1I). Therefore, unlike the amino acid-dependent Dilp regulator(s) predicted by Géminard *et al*. [10], *CCHa2* was found to be primarily sensitive to glucose. Some biological substances are produced by the metabolism of specific nutrients. For example, pyrimidine or purine bases are synthesized from amino acids. Therefore, it is possible that *CCHa2* is down-regulated when glucose is abundant but other nutrients are not available, to limit growth in inhospitable environments. The reduction of *CCHa2* mRNA in TOR-pathway knockdown larvae may recapitulate this scenario (Fig 1J).

In addition to CCHa2, Upd2 was reported to be a glucose-sensitive Dilp regulator expressed in the fat body [9]. The expression of *upd2* in adult flies is up-regulated by feeding with a high-glucose or high-lipid diet. CCHa2 and Upd2, however, responded differently when the TOR pathway was disturbed: whereas *CCHa2* expression was down-regulated in TOR-pathway-knockdown larvae, *upd2* was up-regulated by the inhibition of the TOR pathway in adults [9]. Furthermore, the time course of CCHa2/CCHa2-R signaling is distinct from that of Upd2/Dome signaling. Disruption of *upd2* down-regulated animals’ growth from larval to adult stages [9], whereas *CCHa2-R* mutations reduced growth until late-L3 stages, after which growth was recovered, leading to adults of normal size (Fig 7A). This growth recovery resulted from up-regulation of *dilp6* expression (Fig 8D and 8E), which appears to be a consequence of dysregulated brain Dilps. The lack of growth recovery in *upd2*-knockdown animals in spite of abnormal Dilp production remains unexplained. Nevertheless, these results indicate that *Drosophila melanogaster* possesses multiple insulin regulators that have different nutrient sensitivities. Multi-input Dilp regulation might be advantageous under the imbalanced nutritional conditions that arise in the wild, and this could represent a general strategy for animal growth regulation.

In mammals, different hormones are secreted in response to long-term or short-term metabolic changes. For instance, gut-derived cholecystokinin, glucagon-like peptide-1, and PYY3-36, as well as stomach-derived ghrelin, all of which control feeding behavior, are secreted in response to food ingestion [32]. These hormones respond to acute metabolic changes and immediately signal to the feeding center in the brain. On the other hand, the synthesis or secretion of leptin and adiponectin is affected by the amount of lipid stored in adipocytes [33] [34], suggesting that leptin and adiponectin respond to long-term changes in metabolic status. The expression of *CCHa2* responds to yeast and glucose within 6 hours (Fig 1H), indicating that CCHa2 mediates relatively rapid changes in metabolic status. Thus, it appears that CCHa2 functions as a short-acting metabolic regulator analogous to the mammalian gut- or stomach-derived hormones described above, and that *Drosophila melanogaster* CCHa2 might have an important role in the maintenance of energy homeostasis under volatile nutritional conditions.

### Mechanisms for the regulation of brain Dilps by CCHa2/CCHa2-R signaling

The results from the calcium imaging experiments using brain explants and IPC-specific *CCHa2-R* knockdown strongly suggest that CCHa2 crosses the blood-brain barrier (BBB) to regulate the IPCs, although the underlying mechanism remains elusive. The *Drosophila* BBB consists of two different glial cell layers composed of either the perineurial glia (PG) or the subperineurial glia (SPG) [35] [36]. The SPG cell layer, which is adjacent to the neurons of the brain, forms septate junctions, which function as a barrier to separate the humoral space and the brain, analogously to the mammalian tight junctions formed between endothelial cells. Although several studies have identified important molecules involved in the formation of these septate junctions[36,37] [38] [39], little is known about functional aspects of the BBB [40]. CCHa2 could provide an ideal model for the study of BBB function as well as drug delivery across the BBB.

These experiments also show that peripheral tissue-derived CCHa2 directly activates IPCs in the brain. In mammals, direct sensing of blood glucose levels by pancreatic β-cells is a major trigger for insulin secretion. In these cells, glucose metabolism inhibits the ATP-dependent potassium channel (K_ATP_ channel) and opens voltage-dependent calcium channels (VDCCs), resulting in the exocytosis of insulin-containing granules [41]. The K_ATP_ channel also seems to be involved in insulin secretion in *Drosophila* IPCs [42]. Interestingly, a group of G_α_s- and G_α_q/11-coupled GPCRs can also activate the insulin secretion pathway in mammals [43]. The closest mammalian homologues of CCHa2-R—the Bombesin-related receptor subtypes 3, 1, and 2 (also known as gastrin-releasing-peptide receptor)—signal through G_α_q/11 [44] [45] [46] [47] [48]. The slow rise in [Ca^2+^] in the IPCs in response to CCHa2 application is consistent with CCHa2-R’s mediation of Dilp release through the same pathway.

In contrast to Dilp2, *dilp5* is also regulated by CCHa2/CCHa2-R signaling at the transcriptional level. Although the expression of *dilp5* in the IPCs is activated by the conserved transcription factors Dachshund and Eyeless [27], whether CCHa2-R regulates these factors in IPCs remains unknown.

Overexpression of *CCHa2-R* in IPCs using the GAL4/UAS system displayed inhibitory effects on *dilp5* expression, which prevented us from investigating whether direct CCHa2-R activation in IPCs is sufficient for Dilp regulation. CCHa2-R expression in the brain is not specific to IPCs but occurs in other central neurons (Fig 2B, 2C, 2F and 2G). Therefore, although we have shown that CCHa2-R expression in the IPCs is required for full *dilp5* expression, it is possible that there may also be additional indirect pathways by which CCHa2 may up-regulate the Dilps. Although BBB glial cells are proposed to receive as-yet-unidentified signal(s) from the fat body and re-activate neural stem cells in the brain by secreting Dilp6 [49] [50], *CCHa2-R>nlsGFP* was undetectable in the BBB glial cells (S1G–S1I Fig). Thus BBB cells are unlikely to receive CCHa2 signals or to relay the signals to the IPCs.

### Roles of bombesin family receptor signaling

The closest mammalian homologue of CCHa2-R is *Brs3*, an orphan GPCR, which is a member of the bombesin-like peptide receptor family [51]. *Brs3*-deficient mice develop obesity in association with a reduced metabolic rate and elevated feeding activity [52]. Interestingly, *Brs3* is expressed in pancreatic β-cells both in mice and humans [53]. However, its involvement in insulin regulation has been controversial. Only if *Brs3* knockout adult mice become obese (especially after 23 weeks old) do their plasma insulin levels increase [52]. Since hyper-insulinemia is generally observed in genetically obese mice, the elevation of insulin is most likely the consequence of the obesity rather than the loss of *Brs3* function [52]. On the other hand, a Brs3 agonist promoted insulin secretion in both rodent insulinoma cell lines and in islets isolated from wild-type but not *Brs3* mutants [53]. Our vigorous genetic approach combined with direct observations of Dilp production in IPCs provides the first evidence, to our knowledge, that Bombesin-related receptor signaling activated by its endogenous ligand promotes insulin production.

## Materials and Methods

### Fly strains and diets

The following fly stocks were used: Oregon-R, *y w*, *dilp2*-*GAL4* [12], *Lsp2-GAL4* [13] [54], *ppl-GAL4* [8] [30], *cg-GAL4* [30] [55], *UAS-TSC1/2* [56], *UAS-CCHa2*, *UAS-dilp2* [11], *UAS-CCHa2 RNAi* (VDRC-102257), *UAS-GCaMP6s* [31], *UAS-CCHa2-R-shRNA #3*, *#24*, *#30* (see below), *UAS-dicer1* (see below), and *dilp6* ^*3932*^ [13]. *CCHa2-R* ^*KO51-2*^ and *CCHa2-R* ^*TAL-34*^ are putative null alleles, which were generated by gene targeting using homologous recombination and TALEN, respectively (see below). *CCHa2* ^*CR-1*^, *CCHa2* ^*CR-2*^, and *CCHa2* ^*CR-3*^ were generated using the CRISPR/Cas9 system (see below). *Df(3R)Exel7320* (Exelixis) was used as a deficiency that uncovers *CCHa2*.

Flies were raised at 25°C on regular fly food containing (per liter) 46 g yeast extract, 70 g cornmeal, 100 g glucose, and 6 g agar. For starvation experiments, third-instar larvae (72 hours AEL) were cultured on water agar plates for 18 hours. The starved larvae were later re-fed with different nutrients (yeast paste, 46 g/L peptone, 10% glucose, 0.3% L-isoleucine, or 0.2% L-leucine).

### Mutagenesis

*CCHa2-R* ^*KO51-2*^ was generated by gene targeting using homologous recombination [22] [23]. A 2.4-kb fragment upstream of the second exon and a 2.5-kb fragment downstream of the 6th exon were amplified by PCR and then cloned into the *Nhe*I and *Spe*I sites of the pGX-attP-WN vector [23]. The targeting vector was integrated into the fly genome to generate a donor line. We performed gene targeting as described in Zhou *et al*. (2012) [23] and obtained the *CCHa2-R* ^*KO51-2*^ mutation in which the region from the first methionine through the middle of the 7th transmembrane domain was removed (Figs 3 and S2). *CCHa2-R* ^*TAL-34*^ was generated by inducing double-strand breaks at the *CCHa2* locus using TALEN [24]. A Golden Gate TALEN kit (Addgene) was used to generate two sets of RVD plasmids corresponding to the sequences found in the first coding exon of the *CCHa2-R* gene (1L, 1R, 2L, and 2R; S2 Fig).

1L: TAGTACCGTATGTGCCC

1R: GGAGACGTACATTGTCA

2L: TGCTGTACACGCTCATCTTC

2R: GGCAACGGCACGCTGGTCATCA

For *in vitro* transcription, the RVD fragments were cloned into the pCS2TAL3DDD or pCS2TAL3RRR vector (a gift from K. Hoshijima, The University of Utah). Capped and polyadenylated RNAs were synthesized by *in vitro* transcription from linearized 1L, 1R, 2L, and 2R plasmids, and they were mixed for injection into *y w* embryos. To detect mutated DNA, the target region was amplified by PCR from genomic DNA of mutant candidates, and the amplified fragments were re-annealed. The resulting fragments were digested by T7 endonuclease I (NEB). Sequencing of the mutated fragment revealed that the *CCHa2-R* ^*TAL-34*^ mutation is a 74-bp deletion causing a frame-shift mutation at amino acid position 62 (Figs 3 and S2).

*CCHa2* mutants were generated using the germline-specific CRISPR/Cas9 system as described in Kondo and Ueda (2013) [29] (S5 Fig). The following sgRNA target was used for the mutagenesis of the *CCHa2* gene. Break points of the mutants were determined as described above.

CCHa2 (88): GCCTACGGTCATGTGTGCTACGG

### Vector construction

The *CCHa2-R*-*GAL4*::*p65* construct (Fig 2E) was generated with bacterial artificial chromosome (BAC) recombineering techniques [57] in P[acman] BAC clone CH321-87C13 [58] (Children’s Hospital Oakland Research Institute, Oakland, CA). A landing-site cassette was created by flanking the selectable marker in pSK+-rpsL-kana [59] with 5' *GAL4* and 3' HSP70 UTR arms from pBPGUw [60], a gift of G. Rubin. Fifty-base *CCHa2-R*-specific genomic homology arms were added by PCR using the primers below (genomic homology in lower case, cassette homology in upper case):

CCHa2-R-GAL4-F: 5'-tagaaacaccattgagacatcttgcccaggagcagctccctcctccccacATGAAGCTACTGTCTTCTATCGAACAAGC

CCHa2-R-HSP70-R: 5'-acttccccaccttctgcgggacccccacagtgcgtgatatatccacttacGATCTAAACGAGTTTTTAAGCAAACTCACTCCC

The cassette was recombined into the BAC and then replaced with full-length *GAL4*::*p65-HSP70* amplified from pBPGAL4.2::p65Uw [61] (a gift of G. Rubin) in a second recombination. The final BAC was verified by sequencing the recombined regions and was integrated into the *attP40* site [62] (Genetic Services, Inc., Cambridge, MA).

*UAS-CCHa2* was generated by cloning the coding region of the *CCHa2* gene into the *EcoR*I site of the pUAST vector. Flies were transformed with the *UAS-CCHa2* vector by P-element-mediated transformation to generate the *UAS-CCHa2* stock.

*UAS-CCHa2-R-shRNA* lines were generated as described previously [63]. The following oligonucleotide pairs were annealed and cloned into the *Nhe*I and *Eco*RI sites of the pWALIUM20 vector. The UAS-shRNA constructs were integrated into the *attP40* site [62].

#3 Top strand: 5'-CTAGCAGTGGCTGATCTGTTGGTTATATTTAGTTATATTCAAGCATAAATATAACCAACAGATCAGCCGCG

#3 Bottom strand: 5'-AATTCGCGGCTGATCTGTTGGTTATATTTATGCTTGAATATAACTAAATATAACCAACAGATCAGCCACTG

#24 Top strand: 5'-CTAGCAGTCGATTGTCTACACGCAGGAAATAGTTATATTCAAGCATATTTCCTGCGTGTAGACAATCGGCG

#24 Bottom strand: 5'-AATTCGCCGATTGTCTACACGCAGGAAATATGCTTGAATATAACTATTTCCTGCGTGTAGACAATCGACTG

#30 Top strand: 5'-CTAGCAGTCGAACTGACTTGGAGTTATGTTAGTTATATTCAAGCATAACATAACTCCAAGTCAGTTCGGCG

#30 Bottom strand: 5'-AATTCGCCGAACTGACTTGGAGTTATGTTATGCTTGAATATAACTAACATAACTCCAAGTCAGTTCGACTG

In order to amplify the efficiency of shRNA-mediated gene knockdown, *UAS-dicer1* was constructed. The *dicer1* fragment was amplified by PCR using the *dicer1* cDNA as a template (a gift from Q. Liu, University of Texas Southwestern Medical Center, and Y. Tomari, University of Tokyo). The following primers were used with Q5 DNA polymerase (NEB):

Dcr-1 5': 5'-GGGGTACCAAAATGGCGTTCCACTGGTGCG-3'

Dcr-1 3': 5'-GGAGATCTTAGTCTTTTTTGGCTATCAAGC-3'

The *dicer1* fragment was cloned into the *Kpn*I and *Bgl*II sites of the UASp-K10attB vector (a gift from B. Suter, University of Bern), and the *UAS-dicer1* construct was integrated into the *attP2* site.

### Quantitative RT-PCR

Total RNA from whole larvae or larval tissues was extracted using a PureLink RNA Mini Kit (Life Technologies). cDNAs were prepared by reverse-transcribing 1 μg of total RNA using ReverTra Ace qPCR RT Master Mix with gDNA Remover (Toyobo), and quantitative RT-PCR was performed using Thunderbird qPCR Mix (Toyobo). Expression levels were normalized against those of *rp49*. The following primers were used:

*CCHa2* forward: 5′-AGTGCAGTTGGACTTTGGTAGTGT

*CCHa2* reverse: 5′-AGGGATGCTGTTTAGCATCTATGAC

*CCHa2R* forward: 5′-CTCACTGTCTTTACTGCGGTGAT

*CCHa2R* reverse: 5′-CCACCATGAACTTTGCATACTC

*dilp2* forward: 5′-GTATGGTGTGCGAGGAGTAT

*dilp2* reverse: 5′-TGAGTACACCCCCAAGATAG

*dilp3* forward: 5′-GTCCAGGCCACCATGAAGTTGTGC

*dilp3* reverse: 5′-CTTTCCAGCAGGGAACGGTCTTCG

*dilp5* forward: 5′-TGTTCGCCAAACGAGGCACCTTGG

*dilp5* reverse: 5′-CACGATTTGCGGCAACAGGAGTCG

*dilp6* forward: 5′-TGCTAGTCCTGGCCACCTTGTTCG

*dilp6* reverse: 5′-GGAAATACATCGCCAAGGGCCACC

*rp49* forward: 5′-AGTATCTGATGCCCAACATCG

*rp49* reverse: 5′-CAATCTCCTTGCGCTTCTTG

### Immunostaining and FISH

Antibody staining was conducted as described previously [64], except that the gut samples were fixed in 4% formaldehyde in PBS for 4 hours. Rabbit anti-CCHa2 (1:1,000) [19], rabbit anti-Dilp2 (1:2,500; T. Ida), rabbit anti-Dilp2 (1:2,000, a gift from T. Nishimura, RIKEN Center for Developmental Biology) [27], rabbit anti-Dilp5 (1:2000, a gift from T. Nishimura) [27], mouse anti-Repo c8D12 (1:100; DSHB), mouse anti-GFP (1:500; Invitrogen), and rabbit anti-NPF (1:500; Ray Biotech, Inc) were used as primary antibodies, with anti-mouse-Alexa 488 (1:500; Invitrogen), anti-mouse-Cy3 (1:500; Jackson ImmunoResearch), anti-rabbit-Alexa 488 (1:500; Invitrogen), and anti-rabbit-Cy3 (1:500; Jackson ImmunoResearch) used as secondary antibodies. Alexa 488-conjugated phalloidin (Invitrogen) and TO-PRO-3 (Invitrogen) were used to label the cell membrane and nuclei, respectively. FISH was performed as described in Lehmann and Tautz [65] with a modification designed to amplify signals. Digoxigenin-labeled RNA probes were detected using the TSA Plus Fluorescein Kit (PerkinElmer). For the detection of *CCHa2-R* mRNA, the signals were further amplified using mouse anti-FITC (1:1,000; Jackson ImmunoResearch) and anti-mouse-Alexa 488 antibodies. Images were analyzed by LSM700 confocal microscopy (Zeiss) or DM5000B fluorescence microscopy with a DFC500 CCD camera (Leica).

### Fluorescence quantification

To quantify Dilp2 and Dilp5 levels in IPCs, CNS samples were doubly stained with anti-Dilp2 or anti-Dilp5 and anti-GFP antibodies. Fluorescence images were acquired using a LSM700 confocal microscope (Zeiss). Constant laser power and scan settings were used to image the control and mutant samples. To quantify Dilp levels, Dilp and GFP signal intensities were measured on the same section using the ImageJ software (NIH). Dilp signals were normalized against the GFP signal intensities.

### Calcium imaging

*dilp2-GAL4/UAS-GCaMP6s* and *dilp2-GAL4*, *CCHa2-R* ^*TAL-34*^/*UAS-GCaMP6s*, *CCHa2-R* ^*TAL-34*^ animals were used for imaging. Late-third-instar larvae were dissected in PBS. The ring gland and imaginal disks were removed from the brain. Dissected brains were immersed in 200 μl of PBS and tethered with tungsten wire (0.125 mm diameter, A-M Systems, Inc.) to the bottom of a culture dish (35x10 mm, Falcon). Custom-synthesized CCHa2 (SCRUM Inc.) and synthetic ghrelin and nociception (Peptide institute Inc.) were used. The ligand was pipetted directly into the bath in a volume of 100 μl to yield a final concentration of 10^–9^ M. Imaging was performed with a microscope (Axio Imager.A2, Carl Zeiss) equipped with a spinning disc confocal head (CSU-W1, Yokogawa). We used a 20x water-immersion objective lens (NA = 0.5; Carl Zeiss) mounted with a piezoelectric-activated lens mover (P-725K085 PIFOC, Physik Instrumente GmbH & Co. KG). GCaMP6s signals were excited with a 488-nm laser at 512 x 512 pixel resolution and collected at 250 ms/frame using an EM-CCD camera (ImagEM512, Hamamatsu Photonics) in water-cooled mode. For each IPC within an optical section, regions of interest (ROIs) were selected over multiple IPC somata using the ImageJ software (National Institutes of Health). Raw intensity values for GCaMP6s emission were recorded as mean pixel intensities (value range: 0–65,535) for each ROI at each time point and exported from ImageJ. GCaMP6s signal averaged over 30 frames before stimulation was taken as F_0_, and ΔF/F_0_ was calculated for each time point.

### Analysis of body weight and developmental timing

Larvae were synchronized at hatching (24 hours AEL) and cultured with regular fly food. Ten to thirty-five larvae were collected and weighed at each time point. For the analysis of developmental timing, pupation of synchronized larvae was examined every three hours. Results are shown as the averages of triplicated experiments.

### Feeding assay

The food-ingestion assay was modified from Edgecomb et al. [66]. In brief, 10 larvae were fed with yeast paste containing 1% Brilliant Blue FCF (Wako) for 1 hour or 2 hours. After rinsing with water, larvae were quickly frozen and homogenized in 200 μL of PBS (pH 7.0). The homogenates were centrifuged for 16,000 x g for 10 min, and the supernatants were analyzed spectrophotometrically for absorbance at 625 nm. Results are shown as the averages of triplicated experiments.

### Statistics

The data in each graph were presented as means ± SEM. Two-tailed t-test was used to evaluate the significance of the results between two samples. For multiple comparisons, one-way ANOVA was applied, then pair-wise comparison was performed by Tukey-Kramer (Figs 1H, 4D and 5E and 5F) or Dunnett test (Fig 1I). For the calcium imaging data, Kruskal-Wallis rank sum test was performed for comparison followed by Holm-Bonferroni post-hoc test between four groups of samples (S6 Fig). A significance level of p<0.05 was used for all tests, which was marked by an asterisk in the figure.

## Supporting Information

S1 Fig Expression pattern of *CCHa2-R* in the larval CNS. (A-D) Third-instar larvae expressing *CCHa2-R>nlsGFP* were doubly stained with anti-NPF and anti-GFP antibodies. NPF expression was detected in a subset of *GFP*-positive cells (arrows in B-D). (E, F) *SIFa* mRNA was detected by *in situ* hybridization in third-instar larvae expressing *CCHa2-R>nlsGFP*. GFP was present in all four SIFa-expressing cells located in the anteromedial region of the brain (arrows in F). (G-I) Third-instar larvae expressing *CCHa2-R>nlsGFP* were doubly stained with anti-Repo and anti-GFP antibodies. GFP expression was not detected in the Repo-expressing BBB glial cells (arrowheads in H and I).
(TIF)

S2 Fig *CCHa2-R* mutant alleles. (A) Deletion in the *CCHa2-R* ^*KO51-2*^ mutant allele. Sequences around the break point are shown. (B) TALEN targets for the generation of the *CCHa2-R* ^*TAL-34*^ allele [1L, 1R, 2L, and 2R; underlined]. A mixture of four TALEN mRNAs was injected into early embryos, resulting in a 74-bp deletion between nucleotides 901 and 974 of the *CCHa2-R* gene. (C) Amino acid sequence of the *CCHa2-R* ^*TAL-34*^ mutant protein. The mutant protein has a frame-shift (yellow) leading to a premature termination at the 89th amino acid.
(TIF)

S3 Fig *dilp* mRNA levels in wild-type and *CCHa2-R* mutant brains. (A) Relative amounts of *dilp2*, *-3*, and *-5* mRNA in the wild-type brain were quantified by RT-qPCR. (B) Relative amounts of *dilp3* mRNA in wild-type and *CCHa2-R* mutant brains were quantified by RT-qPCR. Although *dilp3* expression in *CCHa2-R* mutants was lower than that seen in *CCHa2-R* ^*TAL-34*^ */+* control, no significant difference was observed between mutants and *CCHa2-R* ^*KO51-2*^ */+* control, suggesting that *CCHa2-R* is not a specific regulator of *dilp3* transcription.
(TIF)

S4 Fig Feeding activity of *CCHa2-R* mutant larvae. The feeding assay was performed by feeding mid-third-instar larvae with yeast paste containing 1% Brilliant Blue for 1 hour or 2 hours. The larvae were homogenized and the amount of ingested dye was analyzed spectrophotometrically for absorbance at 625 nm.
(TIF)

S5 Fig Generation of *CCHa2* mutant alleles. (A) sgRNA target for the generation of *CCHa2* mutant alleles [CCHa2(88); underlined]. The functional CCHa2 peptide region and PAM sequences are shown in magenta and green, respectively. (B) Deletions in *CCHa2* mutant alleles and the resulting amino acid sequences. All mutant proteins have a frame-shift (yellow) or a deletion within the region of the mature CCHa2 peptide.
(TIF)

S6 Fig Statistical analysis of IPC calcium imaging. The ΔF/F_0_ values before peptide administration (A), 0–2 minutes (B), 2–4 minutes (C), and 4–6 minutes (D) after peptide administration are represented. ΔF/F_0_ value from 5 to 10 different preparations is represented as box plot, and the data distribution is represented as violin plot for each genotype/peptide combination: WT/CCHa2 (n = 10), *CCHa2-R* ^*TAL-34*^ */*CCHa2 (n = 9), WT/Ghrelin (n = 5), and WT/Nociceptin (n = 5).
(TIF)

S7 Fig Knockdown of *CCHa2-R* by shRNAs driven by *CCHa2-R-GAL4*::*p65*. *UAS-CCHa2-R shRNA* lines were crossed with *CCHa2-R-GAL4*::*p65*, and *CCHa2-R* mRNA levels were examined by RT-qPCR. All these shRNAs caused significant reduction in *CCHa2-R* expression.
(TIF)

S1 Movie Calcium imaging of a wild-type brain treated with CCHa2. (AVI)

S2 Movie Calcium imaging of a *CCHa2-R* mutant brain treated with CCHa2. (AVI)

S3 Movie Calcium imaging of a wild-type brain treated with ghrelin. (AVI)

S4 Movie Calcium imaging of a wild-type brain treated with nociceptin. (AVI)
