# Correction: The Nutrient-Responsive Hormone CCHamide-2 Controls Growth by Regulating Insulin-like Peptides in the Brain of *Drosophila melanogaster*

**DOI:** 10.1371/journal.pgen.1005481

**Published:** 2015-09-22

**Authors:** Hiroko Sano, Akira Nakamura, Michael J. Texada, James W. Truman, Hiroshi Ishimoto, Azusa Kamikouchi, Yutaka Nibu, Kazuhiko Kume, Takanori Ida, Masayasu Kojima

In the Materials and Methods section, primer sequences for quantitative RT-PCR of the *CCHa2* gene are incorrect; the correct sequences are as follows.

*CCHa2* forward: GCCTACGGTCATGTGTGCTAC

*CCHa2* reverse: ATCATGGGCAGTAGGCCATT

## References

[1] Sano H, Nakamura A, Texada MJ, Truman JW, Ishimoto H, Kamikouchi A, et al (2015) The Nutrient-Responsive Hormone CCHamide-2 Controls Growth by Regulating Insulin-like Peptides in the Brain of *Drosophila melanogaster* . PLoS Genet 11(5): e1005209 doi:10.1371/journal.pgen.1005209 26020940 10.1371/journal.pgen.1005209 PMC4447355

