# Supplementary figures and images for: The Nutrient-Responsive Hormone CCHamide-2 Controls Growth by Regulating Insulin-like Peptides in the Brain of Drosophila melanogaster
Source: PLoS Genet. 2015 May 28;11(5):e1005209. doi: 10.1371/journal.pgen.1005209 (PMC4447355; doi:10.1371/journal.pgen.1005209)

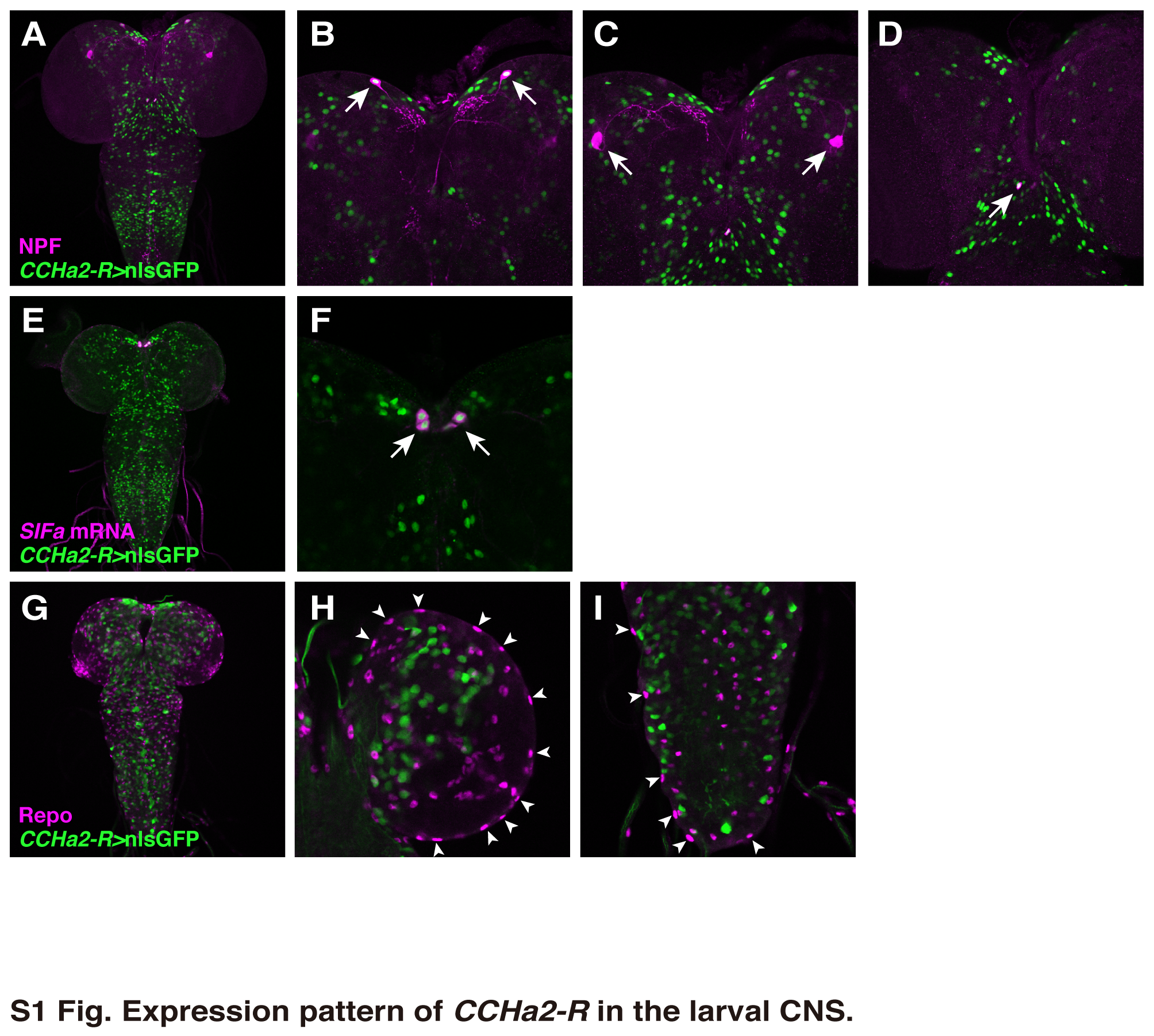

Supplement: S1 Fig — (A-D) Third-instar larvae expressing CCHa2-R>nlsGFP were doubly stained with anti-NPF and anti-GFP antibodies. NPF expression was detected in a subset of GFP-positive cells (arrows in B-D). (E, F) SIFa mRNA was detected by in situ hybridization in third-instar larvae expressing CCHa2-R>nlsGFP. GFP was present in all four SIFa-expressing cells located in the anteromedial region of the brain (arrows in F). (G-I) Third-instar larvae expressing CCHa2-R>nlsGFP were doubly stained with anti-Repo and anti-GFP antibodies. GFP expression was not detected in the Repo-expressing BBB glial cells (arrowheads in H and I). (TIF) [file pgen.1005209.s001.tif]

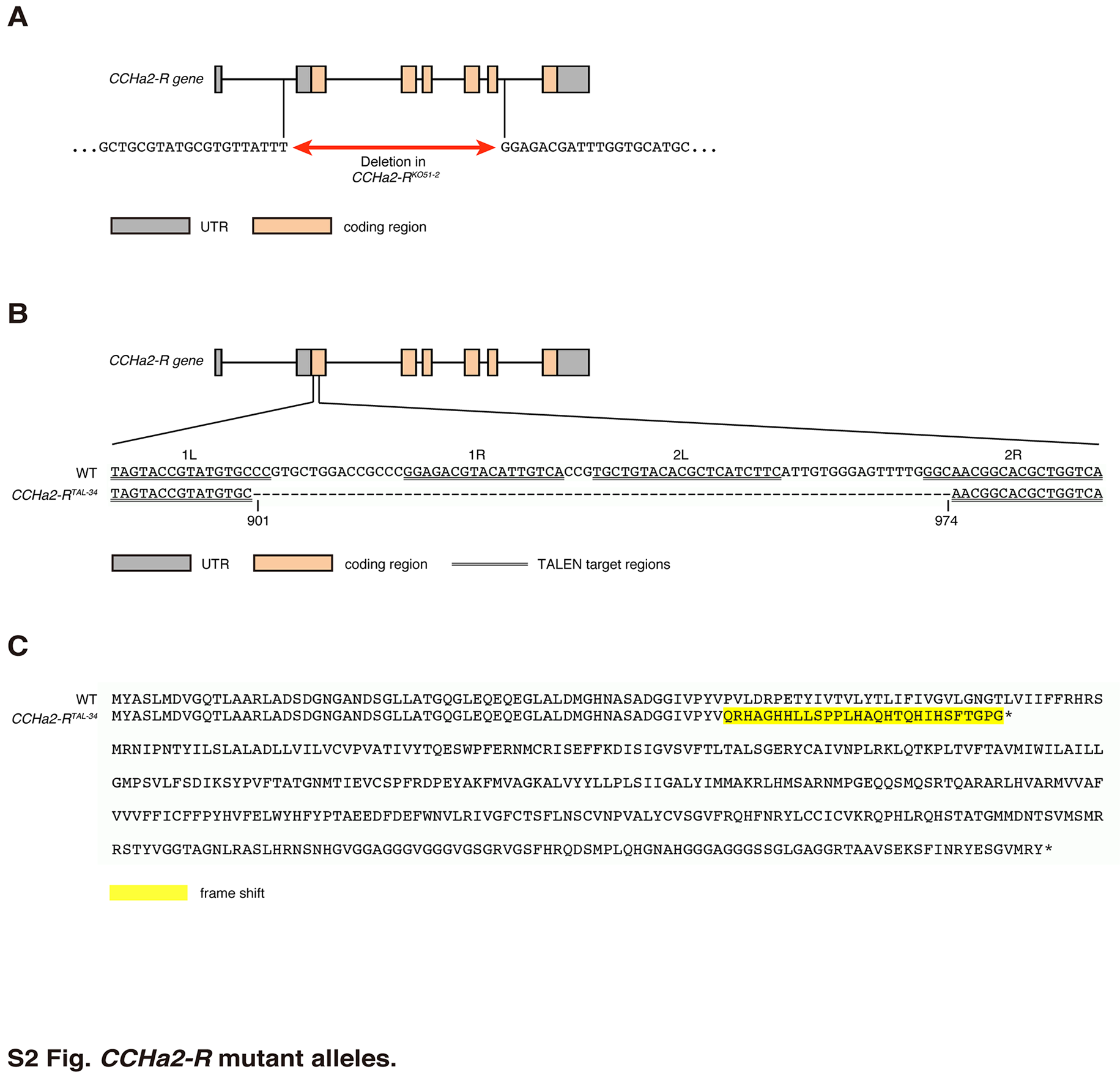

Supplement: S2 Fig — (A) Deletion in the CCHa2-R KO51-2 mutant allele. Sequences around the break point are shown. (B) TALEN targets for the generation of the CCHa2-R TAL-34 allele [1L, 1R, 2L, and 2R; underlined]. A mixture of four TALEN mRNAs was injected into early embryos, resulting in a 74-bp deletion between nucleotides 901 and 974 of the CCHa2-R gene. (C) Amino acid sequence of the CCHa2-R TAL-34 mutant protein. The mutant protein has a frame-shift (yellow) leading to a premature termination at the 89th amino acid. (TIF) [file pgen.1005209.s002.tif]

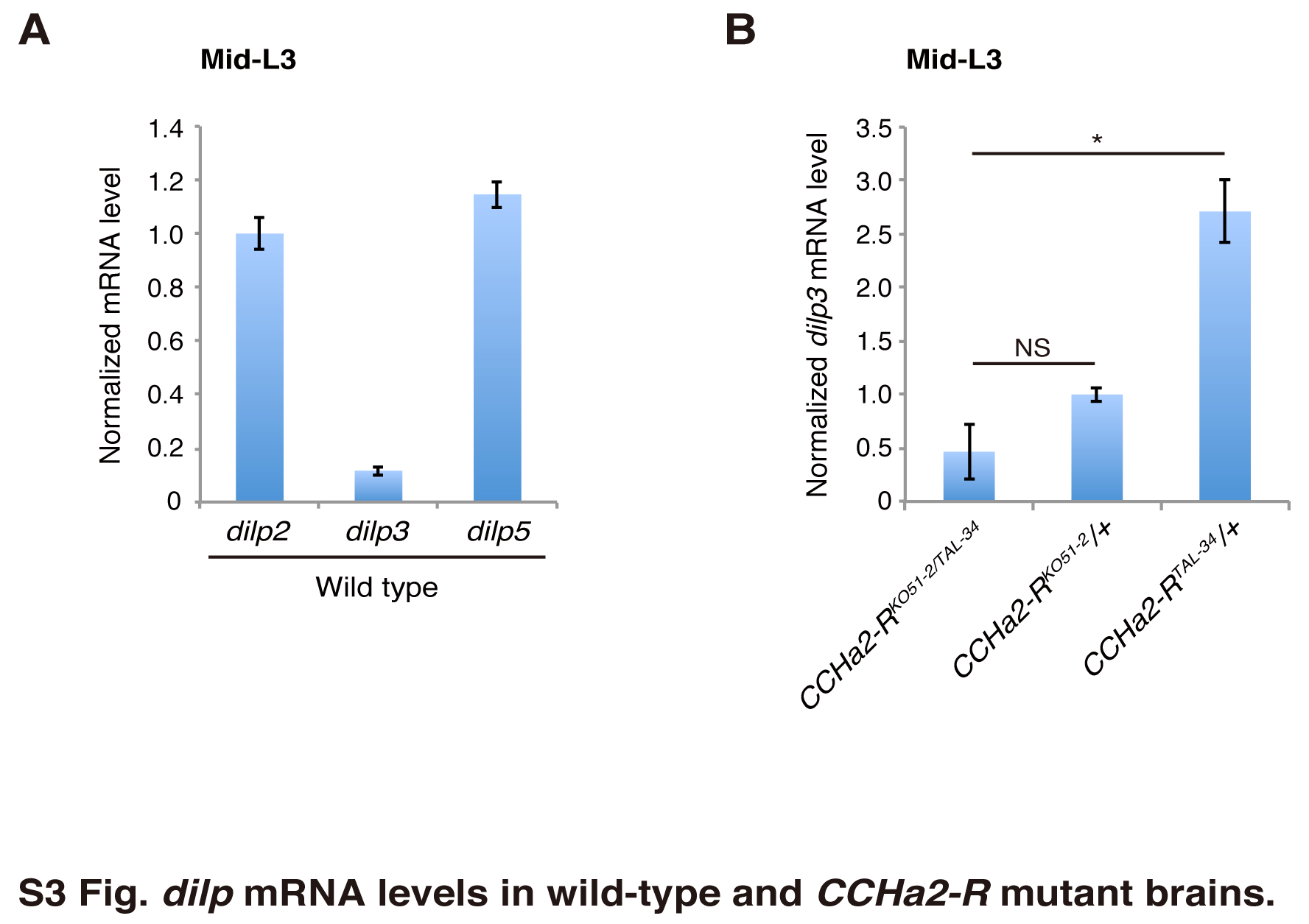

Supplement: S3 Fig — (A) Relative amounts of dilp2, -3, and -5 mRNA in the wild-type brain were quantified by RT-qPCR. (B) Relative amounts of dilp3 mRNA in wild-type and CCHa2-R mutant brains were quantified by RT-qPCR. Although dilp3 expression in CCHa2-R mutants was lower than that seen in CCHa2-R TAL-34 /+ control, no significant difference was observed between mutants and CCHa2-R KO51-2 /+ control, suggesting that CCHa2-R is not a specific regulator of dilp3 transcription. (TIF) [file pgen.1005209.s003.tif]

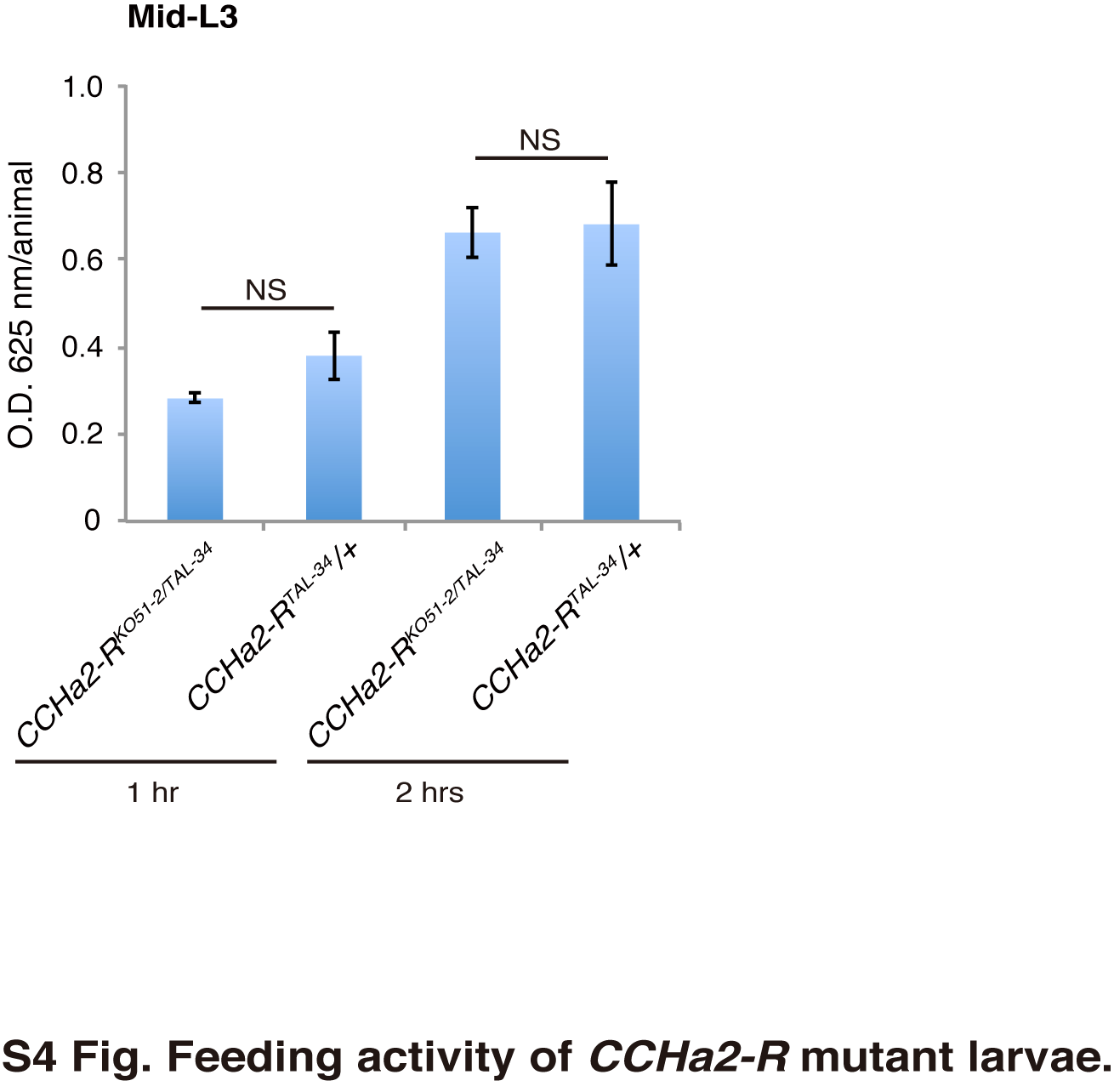

Supplement: S4 Fig — The feeding assay was performed by feeding mid-third-instar larvae with yeast paste containing 1% Brilliant Blue for 1 hour or 2 hours. The larvae were homogenized and the amount of ingested dye was analyzed spectrophotometrically for absorbance at 625 nm. (TIF) [file pgen.1005209.s004.tif]

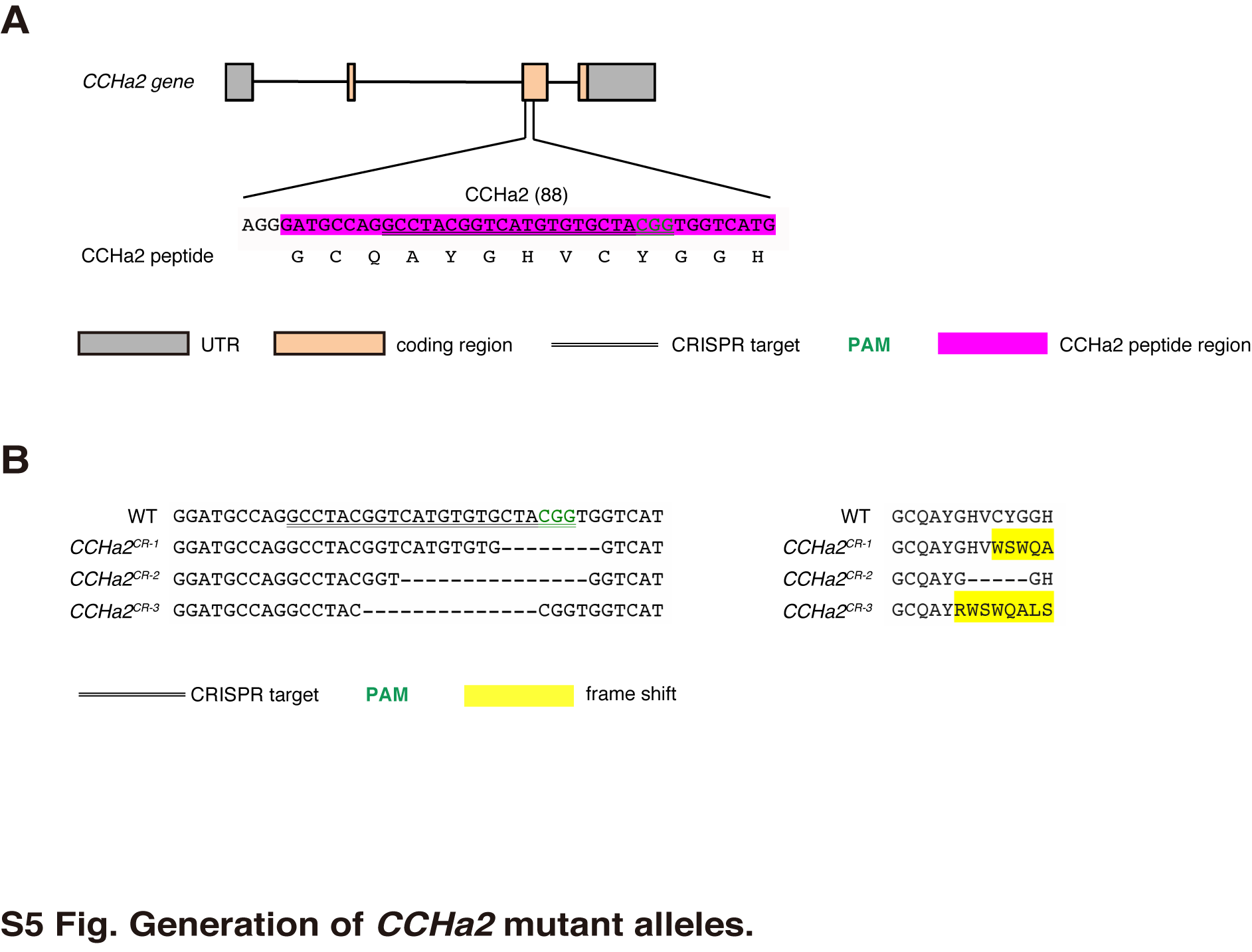

Supplement: S5 Fig — (A) sgRNA target for the generation of CCHa2 mutant alleles [CCHa2(88); underlined]. The functional CCHa2 peptide region and PAM sequences are shown in magenta and green, respectively. (B) Deletions in CCHa2 mutant alleles and the resulting amino acid sequences. All mutant proteins have a frame-shift (yellow) or a deletion within the region of the mature CCHa2 peptide. (TIF) [file pgen.1005209.s005.tif]

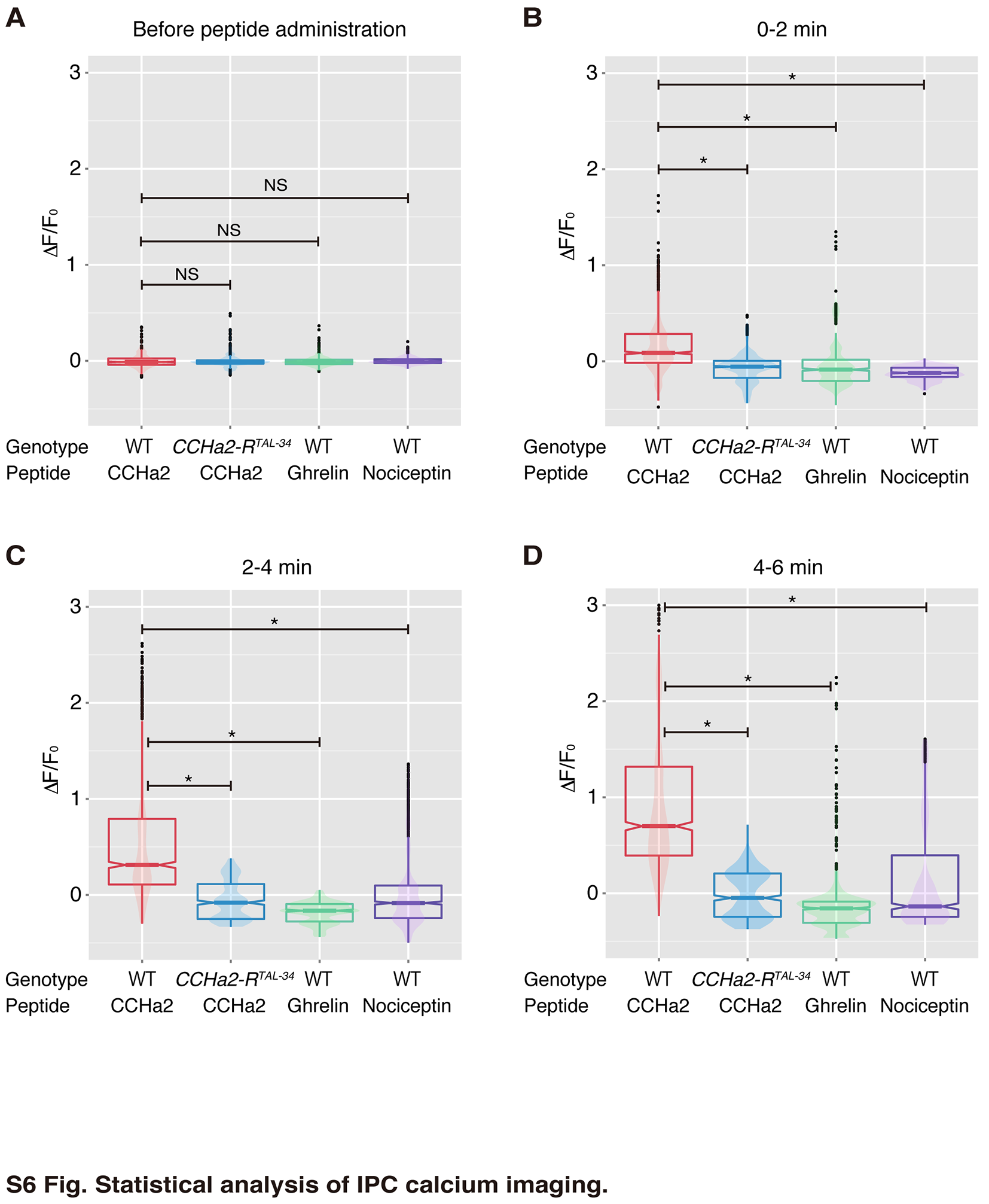

Supplement: S6 Fig — The ΔF/F0 values before peptide administration (A), 0–2 minutes (B), 2–4 minutes (C), and 4–6 minutes (D) after peptide administration are represented. ΔF/F0 value from 5 to 10 different preparations is represented as box plot, and the data distribution is represented as violin plot for each genotype/peptide combination: WT/CCHa2 (n = 10), CCHa2-R TAL-34 /CCHa2 (n = 9), WT/Ghrelin (n = 5), and WT/Nociceptin (n = 5). (TIF) [file pgen.1005209.s006.tif]

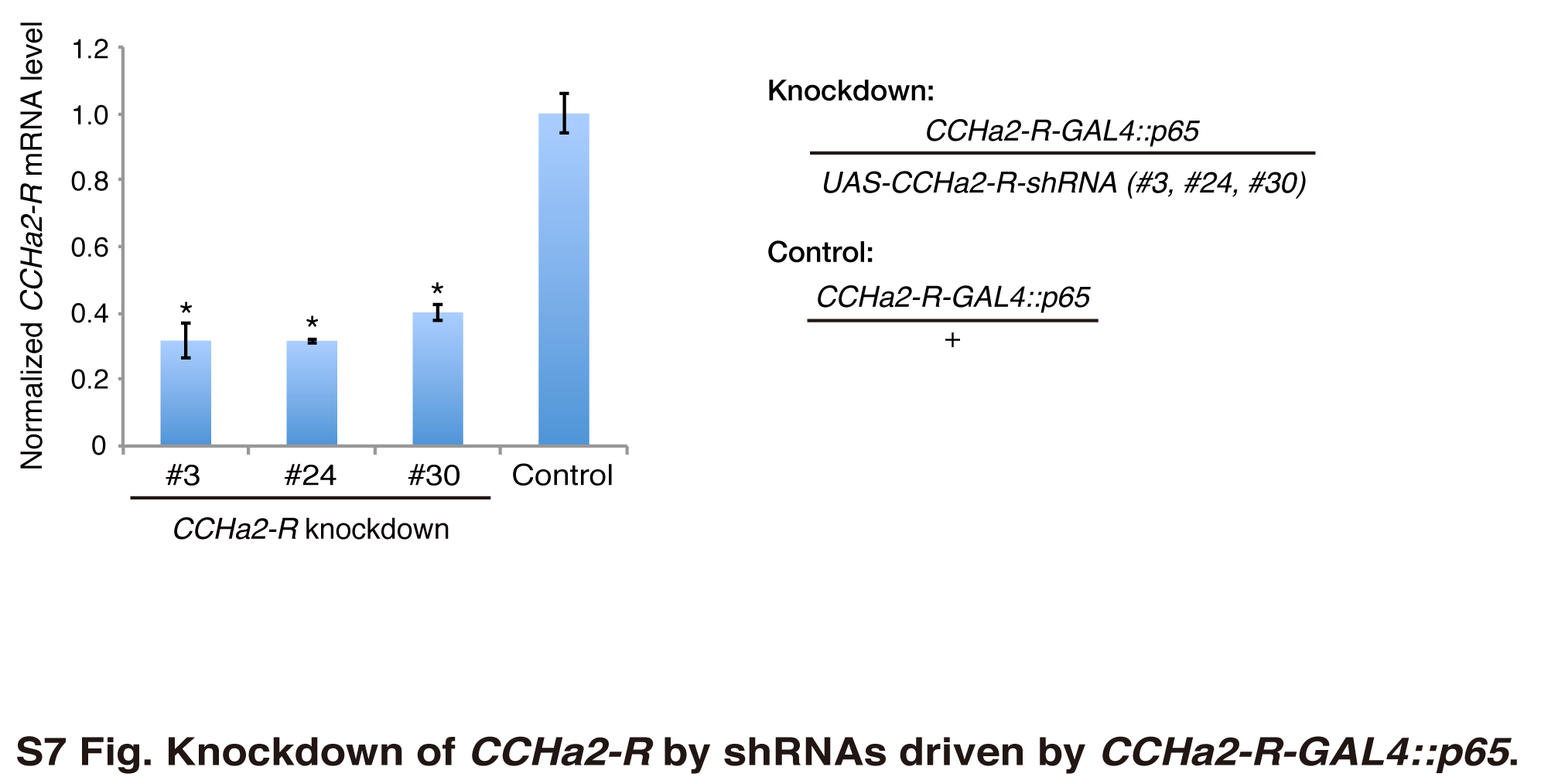

Supplement: S7 Fig — UAS-CCHa2-R shRNA lines were crossed with CCHa2-R-GAL4::p65, and CCHa2-R mRNA levels were examined by RT-qPCR. All these shRNAs caused significant reduction in CCHa2-R expression. (TIF) [file pgen.1005209.s007.tif]
